# Global, regional and national burdens of reproduction-related congenital birth defects, 1990–2019

**DOI:** 10.3389/fpubh.2024.1328282

**Published:** 2024-02-26

**Authors:** Lin Shen, Jie Li, Hanwang Zhang, Yiqing Zhao

**Affiliations:** Department of Reproductive Medicine, Tongji Hospital, Tongji Medical College, Huazhong University of Science and Technology, Wuhan, China

**Keywords:** congenital birth defects, Klinefelter syndrome, Turner syndrome, urogenital congenital anomalies, global burden of disease

## Abstract

**Background:**

Reproduction-related congenital birth defects (RCBDs), including Klinefelter syndrome (KS), Turner syndrome (TS), and urogenital congenital anomalies (UCA), can lead to severe physical and psychosocial disorders. The global impact of RCBDs on children and adults is unknown, which limits high-quality development of populations and increases in life expectancy *per capita*.

**Methods:**

Annual incidence rates, prevalence rates (PR), and disability-adjusted life year (DALY) rates were collected for KS, TS, and UCA for 204 countries and territories, including at birth, for children younger than 1 year, and age-standardized (AS) for all ages. Linear regression was used to calculate their estimated annual percentage changes (EAPCs). Finally, the relationships between EAPCs of each indicator and sociodemographic index (SDI) was investigated using Pearson correlation analysis.

**Results:**

Globally, the age-standardized prevalence rate (ASPR) trend is decreasing in KS and TS and increasing in UCA. The DALY rates for children younger than 1 year were on a downward trend in KS and UCA, while they were still rising for TS. The AS-DALY rates were all on a downward trend in KS, TS, and UCA. The DALY rates of KS, TS and UCA were found higher in high-income countries in North America. In addition, the burdens of TS and UCA went down with increasing SDI, whereas the burden of KS increased with increasing SDI.

**Conclusion:**

The global burdens of RCBDs have decreased since 1990. This finding can help policymakers implement cost-effective interventions to reduce the burdens of RCBDs.

## Introduction

Congenital birth defects are important parts of the global burden of disease, seriously threatening the lives and health of children. They were estimated to be the 4th leading cause of death among children under 5 years of age in 2019 (1). Reproduction-related congenital birth defects (RCBDs), a subset of congenital birth defects including Klinefelter syndrome (KS), Turner syndrome (TS), and urogenital congenital anomalies (UCA), can lead to infertility and malformations of reproduction-related organs, and even psychosocial disorders. The global impact of RCBDs on children and even adults is unknown.

To understand the changing health challenges, promote accountability, and improve lives worldwide facing people across the world in the 21st century, the Institute for Health Metrics and Evaluation (IHME) conducts the Global Burden of Diseases, Injuries, and Risk Factors (GBD) study, the most comprehensive global observational epidemiological study, by tracking progress within and between countries. The GBD study contains country-level results, uncertainty quantification, and data that are comparable across geographies, over time, and across health status. It is a way to quantify health loss due to hundreds of diseases, injuries and risk factors in order to improve health systems and eliminate disparities. The 2019 GBD Study generates updated estimates of global prevalence and disability-adjusted life year (DALY) rates of RCBDs. Here, GBD 2019 was investigated for the first time to determine global, regional, and national prevalence and DALY rates of KS, TS, and UCA, as well as by age, sex, and sociodemographic index (SDI) from 1990 to 2019 to provide a comprehensive and comparable analysis of the burdens of RCBDs.

## Methods

### Data sources

Data on the burdens of RCBDs in 204 countries and territories from 1990 to 2019 were obtained from the Global Health Data Exchange (GHDE)^1^ (data access, August 14, 2023) (2). GHDE is the public data platform where IHME places the aggregated GBD results. Developed and calculated by GBD researchers, these estimates of the SDI, a composite indicator of development status that includes *per capita* income, years of schooling, and the fertility rate of women under 25, are strongly correlated with health outcomes. It ranges from 0 to 1, with higher values being better. The 204 countries and territories are divided by SDI quintiles into five regions (low SDI, low-middle SDI, middle SDI, middle-high SDI, and high SDI) and geographically into 21 GBD regions. Previous studies have detailed the general methods for GBD 2019 and estimated the burdens of congenital birth defects (Supplementary methods) (3–5).

### Case definitions

The definition of congenital anomalies in GBD cases includes any condition resulting from abnormal embryonic development at birth, excluding those directly caused by infection or drug abuse (e.g., fetal alcohol syndrome, congenital syphilis) and minor anomalies as defined by European surveillance of congenital anomalies (5). Further, the case definition of GBD includes only live births and excludes infants terminated after prenatal diagnosis and stillbirths (5).

KS is due to the inheritance of one or more extra X chromosomes from the paternal or (and) maternal side, with the most common karyotype being “47, XXY” (6, 7). Typical clinical features include male infertility, small testes, hypergonadotropic hypogonadism, and gynecomastia (6, 7). TS refers to the deletion or structural abnormality of 1 X chromosome in females, often presenting as mosaic karyotypes (8–10). It is associated with female infertility, short stature, delayed puberty, ovarian hypoplasia, and hypogonadotropic hypogonadism (8). In addition, there are many types of UCA, such as polycystic kidneys, double ureters, bladder exstrophy, double uterus, and hermaphroditism.

### Data analysis

This study characterized the burdens of RCBDs (KS, TS, UCA) by age, sex, year, and location. Age-standardized annual incidence, prevalence, and DALY rates were collected for KS, TS, and UCA at birth, for children younger than 1 year, and for all ages. KS and TS occur only in males and females, respectively; therefore, non-onset sex was not considered in the analyses. Congenital birth defects occur at birth and, by definition, there are no postnatal cases. Therefore, the indicators were simplified to prevalence rate (PR) at birth (PR at birth is equivalent to incidence at birth), PR for children younger than 1 year, age-standardized prevalence rate (ASPR), DALY rate for children younger than 1 year, and age-standardized DALY (AS-DALY) rate. In addition, linear regression was used to calculate their estimated annual percentage changes (EAPCs) to assess long-term trends. Finally, the relationships between EAPCs of each indicator and SDI was investigated using Pearson correlation analysis. All statistical analyses were performed using R (version 4.3.1). The threshold for two-sided *p* values was less than 0.05.

## Results

### Global prevalence trends of RCBDs from 1990 to 2019

Results from this study indicates that, the global PR at birth for KS was 21.9 per 100,000, the global PR for children younger than 1 year was 21.0 per 100,000, and the global ASPR was 6.7 per 100,000 in 2019 (Table 1). The global ASPR declined, with an EAPC of −0.82 from 1990 to 2019 (Table 1; Supplementary Figure S1). Similarly, PR at birth and PR for children younger than 1 year also showed a declining trend (Table 1). The PR of KS was highest in the high SDI region, about 4 times the global level (Table 1; Supplementary Figure S2). The PR in the high SDI region had remained stable over 30 years, including PR at birth, PR for children younger than 1 year and ASPR (Table 1; Supplementary Figure S1). The other four SDI regions showed a decreasing trend in PR in all 3 age strata (Table 1; Supplementary Figure S1). PR at birth and for children younger than 1 year remained on an upward trend in Central Europe, High-income Asia Pacific, Oceania (highest), South Asia, and Southeast Asia (Table 1; Supplementary Figure S1). There were 7 regions with upward trends in ASPR, including Central Europe, high-income Asia-Pacific, Oceania, South Asia, South-East Asia, Tropical Latin America, and Western Europe (Table 1; Supplementary Figure S1).

**Table 1:** Prevalence rate (PR) for Klinefelter syndrome in 1990 and 2019 and their estimated annual percent change (EAPCs).

KS (per 100,000)	PR at birth			PR for children younger than 1 year			ASPR		
	1990 (95% UI)	2019 (95% UI)	EAPC (1990–2019, 95%CI)	1990 (95% UI)	2019 (95% UI)	EAPC (1990–2019, 95%CI)	1990 (95% UI)	2019 (95% UI)	EAPC (1990–2019, 95%CI)
Global	25.6 (18.3–35.3)	21.9 (15.6–30.3)	−0.58 (−0.65 to −0.52)	24.7 (17.6–33.8)	21.0 (15.0–29.0)	−0.60 (−0.67 to −0.54)	8.4 (6.2–10.7)	6.7 (4.9–8.7)	−0.82 (−0.86 to −0.79)
**Sex**									
Female	0.0 (0.0–0.0)	0.0 (0.0–0.0)	0.00 (0.00 to 0.00)	0.0 (0.0–0.0)	0.0 (0.0–0.0)	0.00 (0.00 to 0.00)	0.0 (0.0–0.0)	0.0 (0.0–0.0)	0.00 (0.00 to 0.00)
Male	25.6 (18.3–35.3)	21.9 (15.6–30.3)	−0.58 (−0.65 to −0.52)	24.7 (17.6–33.8)	21.0 (15.0–29.0)	−0.60 (−0.67 to −0.54)	8.4 (6.2–10.7)	6.7 (4.9–8.7)	−0.82 (−0.86 to −0.79)
**SDI region**									
Low	9.6 (6.2–13.7)	9.3 (6.0–13.3)	−0.10 (−0.12 to −0.07)	9.0 (5.8–12.9)	8.8 (5.6–12.5)	−0.10 (−0.13 to −0.07)	1.7 (1.1–2.4)	1.6 (1.0–2.3)	−0.13 (−0.15 to −0.10)
Low-middle	16.2 (10.8–22.7)	14.9 (10.1–20.9)	−0.31 (−0.36 to −0.26)	15.3 (10.3–21.3)	14.1 (9.5–19.7)	−0.32 (−0.38 to −0.27)	3.2 (2.2–4.4)	2.8 (1.9–3.9)	−0.52 (−0.56 to −0.48)
Middle	28.1 (19.5–39.4)	23.0 (16.0–32.2)	−0.72 (−0.81 to −0.63)	26.6 (18.5–37.3)	21.8 (15.3–30.5)	−0.72 (−0.80 to −0.63)	6.3 (4.5–8.3)	5.1 (3.6–6.7)	−0.82 (−0.89 to −0.76)
Middle-high	26.9 (19.0–37.1)	25.7 (18.2–35.6)	−0.20 (−0.24 to −0.16)	25.7 (18.3–35.5)	24.7 (17.4–34.1)	−0.18 (−0.22 to −0.14)	8.5 (6.3–11.0)	7.9 (5.8–10.2)	−0.21 (−0.25 to −0.17)
High	77.3 (56.7–102.8)	82.5 (59.9–111.7)	0.01 (−0.11 to 0.14)	74.0 (54.5–98.0)	78.7 (57.2–106.4)	0.01 (−0.11 to 0.12)	28.0 (21.1–35.1)	28.9 (21.6–36.5)	0.02 (−0.02 to 0.05)
**GBD region**									
Andean Latin America	26.7 (18.1–37.6)	17.3 (11.9–24.0)	−1.71 (−1.84 to −1.58)	25.3 (17.0–35.3)	16.4 (11.3–22.8)	−1.71 (−1.84 to −1.58)	5.1 (3.6–6.8)	3.9 (2.7–5.2)	−1.12 (−1.21 to −1.02)
Australasia	30.3 (21.7–42.2)	26.2 (18.9–35.6)	−0.47 (−0.55 to −0.39)	29.3 (20.9–40.8)	25.5 (18.3–34.5)	−0.46 (−0.54 to −0.38)	8.2 (5.9–10.6)	8.2 (6.0–10.5)	−0.04 (−0.11 to 0.03)
Caribbean	28.2 (19.3–39.5)	27.4 (18.5–39.3)	−0.45 (−0.57 to −0.33)	26.3 (18.0–36.8)	25.7 (17.4–36.9)	−0.44 (−0.56 to −0.31)	5.8 (4.1–7.7)	5.8 (4.2–7.7)	−0.17 (−0.25 to −0.10)
Central Asia	29.4 (20.4–41.7)	30.7 (21.3–43.2)	−0.10 (−0.31 to 0.10)	27.7 (19.1–39.1)	28.9 (20.0–40.6)	−0.10 (−0.31 to 0.10)	6.2 (4.5–8.3)	6.4 (4.6–8.7)	−0.04 (−0.20 to 0.13)
Central Europe	59.3 (42.0–81.6)	59.9 (43.0–81.9)	0.37 (0.29 to 0.44)	56.4 (40.0–77.2)	57.1 (41.1–78.3)	0.39 (0.31 to 0.48)	16.0 (11.8–20.8)	17.2 (12.8–22.0)	0.52 (0.29 to 0.76)
Central Latin America	31.8 (22.0–44.6)	20.8 (14.7–28.9)	−1.63 (−1.69 to −1.58)	30.1 (20.7–41.8)	19.7 (13.9–27.3)	−1.62 (−1.67 to −1.57)	6.4 (4.5–8.6)	5.2 (3.7–6.8)	−0.80 (−0.82 to −0.77)
Central Sub-Saharan Africa	8.4 (5.4–12.4)	8.5 (5.4–12.1)	0.02 (−0.01 to 0.05)	7.9 (5.0–11.6)	8.0 (5.0–11.4)	0.01 (−0.02 to 0.04)	1.4 (0.9–2.0)	1.4 (0.9–2.0)	−0.09 (−0.12 to −0.06)
East Asia	37.9 (26.8–52.2)	34.2 (24.1–48.2)	−0.43 (−0.46 to −0.39)	35.9 (25.4–49.5)	32.4 (22.8–45.5)	−0.42 (−0.46 to −0.38)	9.0 (6.6–11.7)	7.9 (5.8–10.4)	−0.55 (−0.63 to −0.48)
Eastern Europe	8.0 (5.4–11.2)	9.4 (6.4–13.3)	0.11 (−0.47 to 0.69)	7.6 (5.1–10.6)	8.9 (6.0–12.5)	0.13 (−0.45 to 0.71)	1.9 (1.3–2.6)	2.2 (1.5–3.0)	0.17 (−0.34 to 0.68)
Eastern Sub-Saharan Africa	8.2 (5.2–11.8)	8.1 (5.1–11.7)	−0.08 (−0.14 to −0.03)	7.7 (4.9–11.1)	7.6 (4.8–11.1)	−0.08 (−0.14 to −0.03)	1.4 (0.9–2.0)	1.4 (0.9–2.0)	−0.07 (−0.11 to −0.02)
High-income Asia Pacific	19.7 (14.0–26.6)	22.7 (16.8–30.2)	0.52 (0.49 to 0.56)	19.0 (13.5–25.5)	21.8 (16.1–28.8)	0.54 (0.49 to 0.58)	7.9 (5.8–10.1)	8.4 (6.3–10.6)	0.34 (0.27 to 0.41)
High-income North America	112.0 (80.4–150.9)	117.0 (84.5–160.5)	−0.28 (−0.46 to −0.10)	107.1 (77.0–144.2)	111.3 (80.5–151.7)	−0.30 (−0.47 to −0.12)	41.2 (30.7–52.1)	40.8 (30.1–52.6)	−0.20 (−0.26 to −0.13)
North Africa and Middle East	7.1 (4.6–10.3)	5.7 (3.8–8.2)	−1.07 (−1.17 to −0.97)	6.7 (4.3–9.6)	5.4 (3.6–7.7)	−1.06 (−1.16 to −0.96)	1.4 (0.9–1.9)	1.3 (0.9–1.8)	−0.26 (−0.31 to −0.22)
Oceania	34.5 (23.3–49.2)	43.1 (28.5–61.8)	0.84 (0.77 to 0.90)	32.4 (21.8–46.0)	40.5 (26.6–57.8)	0.84 (0.77 to 0.90)	6.5 (4.5–8.8)	7.4 (5.1–10.3)	0.46 (0.42 to 0.49)
South Asia	7.5 (4.8–10.8)	7.7 (4.8–11.0)	0.09 (0.07 to 0.11)	7.0 (4.5–10.1)	7.2 (4.6–10.4)	0.10 (0.07 to 0.12)	1.4 (0.9–1.9)	1.4 (0.9–2.0)	0.04 (0.02 to 0.06)
Southeast Asia	33.0 (22.4–46.0)	35.5 (24.3–49.6)	0.35 (0.29 to 0.41)	31.1 (21.2–43.3)	33.6 (22.9–46.7)	0.37 (0.30 to 0.43)	6.5 (4.6–8.7)	6.9 (4.8–9.2)	0.23 (0.20 to 0.26)
Southern Latin America	6.9 (4.6–9.6)	6.5 (4.3–9.3)	−0.19 (−0.31 to −0.07)	6.5 (4.3–9.0)	6.1 (4.0–8.8)	−0.19 (−0.31 to −0.07)	1.8 (1.2–2.5)	1.7 (1.1–2.3)	−0.34 (−0.46 to −0.22)
Southern Sub-Saharan Africa	6.2 (4.2–8.8)	6.1 (4.0–8.7)	−0.21 (−0.37 to −0.04)	5.9 (3.9–8.4)	5.8 (3.8–8.2)	−0.20 (−0.36 to −0.05)	1.3 (0.9–1.9)	1.3 (0.9–1.9)	−0.13 (−0.17 to −0.09)
Tropical Latin America	55.7 (37.3–80.3)	54.2 (37.9–75.4)	−0.13 (−0.17 to −0.09)	52.5 (35.3–75.3)	51.1 (35.7–71.0)	−0.13 (−0.16 to −0.09)	10.7 (7.5–14.5)	11.9 (8.5–15.7)	0.46 (0.43 to 0.48)
Western Europe	99.2 (74.0–129.7)	96.9 (70.9–130.4)	−0.07 (−0.11 to −0.02)	94.6 (70.5–123.3)	92.8 (68.0–124.2)	−0.06 (−0.10 to −0.01)	36.4 (27.8–45.2)	37.2 (27.8–46.5)	0.11 (0.09 to 0.14)
Western Sub-Saharan Africa	7.6 (4.8–11.0)	7.6 (4.9–11.0)	−0.04 (−0.12 to 0.04)	7.2 (4.5–10.3)	7.2 (4.6–10.4)	−0.05 (−0.13 to 0.03)	1.4 (0.9–1.9)	1.4 (0.9–1.9)	−0.09 (−0.14 to −0.05)

KS, Klinefelter syndrome; ASPR, age-standardized prevalence rate.

The global PR at birth for TS was 53.1 per 100,000, 49.2 per 100,000 for children younger than 1 year, and 21.6 per 100,000 for ASPR in 2019 (Table 2). The global PR at birth and for children younger than 1 year were still increasing (EAPC = 0.06, 0.06, respectively), and ASPR was decreasing (EAPC = −0.16) (Table 2; Supplementary Figure S1). The highest PR for TS was in the high SDI region, approximately 1.5 times the global level, with decreasing PR in all 3 age strata (Table 1; Supplementary Figure S2). In the low-middle SDI region, PR was increasing in all 3 age strata (Table 1; Supplementary Figure S1). High-income Asia Pacific also had the highest PR for TS, and its PR trend was downward (EAPC = −0.32 at birth, −0.29 for children younger than 1 year, −0.16 for ASPR). Oceania had the greatest upward trend in PR (EAPC = 0.80 at birth, 0.80 for children younger than 1 year, 0.38 for ASPR).

**Table 2:** Prevalence rate (PR) for Turner syndrome in 1990 and 2019 and their estimated annual percent change (EAPCs).

TS (per 100,000)	PR at birth			PR for children younger than 1 year			ASPR		
	1990 (95% UI)	2019 (95% UI)	EAPC (1990–2019, 95%CI)	1990 (95% UI)	2019 (95% UI)	EAPC (1990–2019, 95%CI)	1990 (95% UI)	2019 (95% UI)	EAPC (1990–2019, 95%CI)
Global	51.1 (35.3–68.7)	53.1 (36.7–71.5)	0.06 (0.01 to 0.11)	51.0 (35.4–68.5)	49.2 (34.2–66.1)	0.06 (0.01 to 0.11)	22.6 (16.5–29.1)	21.6 (15.8–28.2)	−0.16 (−0.16 to −0.15)
**Sex**									
Female	51.1 (35.3–68.7)	53.1 (36.7–71.5)	0.06 (0.01 to 0.11)	51.0 (35.4–68.5)	49.2 (34.2–66.1)	0.06 (0.01 to 0.11)	22.6 (16.5–29.1)	21.6 (15.8–28.2)	−0.16 (−0.16 to −0.15)
Male	0.0 (0.0–0.0)	0.0 (0.0–0.0)	0.00 (0.00 to 0.00)	0.0 (0.0–0.0)	0.0 (0.0–0.0)	0.00 (0.00 to 0.00)	0.0 (0.0–0.0)	0.0 (0.0–0.0)	0.00 (0.00 to 0.00)
**SDI region**									
Low	64.5 (42.9–88.6)	64.7 (43.0–89.1)	−0.05 (−0.08 to −0.02)	62.2 (41.4–85.7)	61.9 (41.4–85.1)	−0.05 (−0.08 to −0.01)	19.2 (13.6–25.6)	19.2 (13.7–25.6)	−0.03 (−0.05 to −0.01)
Low-middle	53.8 (35.9–73.3)	55.1 (37.0–75.4)	0.09 (0.05 to 0.13)	52.8 (35.5–72.3)	51.5 (34.4–70.2)	0.09 (0.05 to 0.13)	17.1 (12.4–22.8)	17.7 (12.7–23.4)	0.12 (0.11 to 0.14)
Middle	40.2 (27.7–53.7)	39.0 (27.2–52.0)	−0.16 (−0.25 to −0.07)	37.5 (26.2–50.3)	38.6 (26.7–51.5)	−0.15 (−0.24 to −0.06)	15.8 (11.6–20.6)	16.7 (12.2–21.7)	0.22 (0.19 to 0.25)
Middle-high	32.5 (23.0–42.9)	30.4 (21.6–40.1)	−0.34 (−0.39 to −0.29)	29.6 (21.1–39.2)	31.7 (22.4–41.8)	−0.33 (−0.38 to −0.29)	18.2 (13.5–23.6)	16.8 (12.5–21.8)	−0.29 (−0.32 to −0.25)
High	88.1 (63.8–114.4)	86.2 (62.5–112.0)	−0.13 (−0.17 to −0.09)	84.5 (61.4–109.8)	86.6 (62.9–112.8)	−0.13 (−0.17 to −0.10)	53.0 (39.5–67.9)	50.0 (37.0–63.8)	−0.22 (−0.24 to −0.21)
**GBD region**									
Andean Latin America	46.7 (31.8–64.0)	31.4 (21.7–42.4)	−1.53 (−1.65 to −1.41)	30.3 (21.0–40.7)	45.0 (30.7–61.8)	−1.53 (−1.64 to −1.41)	16.5 (11.9–21.9)	13.5 (9.9–17.7)	−0.76 (−0.83 to −0.70)
Australasia	44.7 (32.4–58.7)	40.9 (29.4–54.0)	−0.28 (−0.32 to −0.25)	40.5 (29.2–53.7)	44.7 (32.3–58.3)	−0.31 (−0.34 to −0.28)	24.1 (17.9–31.5)	23.6 (17.2–30.8)	−0.08 (−0.11 to −0.06)
Caribbean	42.6 (29.0–57.5)	39.5 (27.0–53.5)	−0.44 (−0.51 to −0.37)	37.8 (25.9–51.2)	40.5 (27.6–54.6)	−0.42 (−0.49 to −0.35)	16.3 (11.8–21.3)	15.6 (11.4–20.5)	−0.23 (−0.27 to −0.19)
Central Asia	40.2 (27.7–54.4)	37.3 (25.5–50.1)	−0.53 (−0.63 to −0.43)	35.9 (24.6–48.3)	38.6 (26.6–52.2)	−0.52 (−0.62 to −0.42)	16.2 (11.6–21.3)	15.6 (11.2–20.4)	−0.25 (−0.30 to −0.20)
Central Europe	38.1 (27.4–50.2)	30.1 (21.9–39.2)	−0.66 (−0.96 to −0.37)	29.5 (21.4–38.5)	37.1 (26.8–48.8)	−0.63 (−0.92 to −0.35)	20.9 (15.5–27.1)	18.6 (13.7–24.1)	−0.27 (−0.41 to −0.13)
Central Latin America	108.1 (75.3–142.3)	90.5 (64.9–118.8)	−0.68 (−0.72 to −0.64)	87.7 (63.2–115.4)	103.9 (72.9–137.1)	−0.63 (−0.68 to −0.59)	45.1 (33.5–57.7)	46.5 (34.8–59.5)	0.09 (0.06 to 0.11)
Central Sub-Saharan Africa	63.4 (42.5–88.2)	67.1 (44.9–93.0)	0.19 (0.14 to 0.24)	64.4 (43.3–88.7)	60.9 (40.9–84.9)	0.19 (0.13 to 0.24)	19.0 (13.4–25.4)	19.7 (13.8–26.3)	0.09 (0.05 to 0.12)
East Asia	23.6 (16.5–31.7)	25.1 (17.7–33.5)	0.51 (0.40 to 0.62)	24.3 (17.2–32.5)	22.9 (16.1–30.8)	0.50 (0.39 to 0.61)	12.0 (8.8–15.6)	12.6 (9.4–16.4)	0.30 (0.26 to 0.34)
Eastern Europe	22.3 (15.6–29.8)	21.0 (14.7–28.1)	−0.55 (−0.68 to −0.42)	20.4 (14.3–27.2)	21.7 (15.2–29.1)	−0.53 (−0.65 to −0.40)	11.2 (8.1–14.8)	10.9 (7.9–14.3)	−0.25 (−0.33 to −0.17)
Eastern Sub-Saharan Africa	64.5 (42.9–88.0)	63.2 (42.4–86.9)	−0.12 (−0.16 to −0.08)	60.7 (40.8–83.7)	61.9 (41.2–84.6)	−0.12 (−0.16 to −0.08)	19.4 (13.7–25.8)	19.3 (13.9–25.6)	−0.05 (−0.07 to −0.03)
High-income Asia Pacific	115.0 (83.8–149.1)	109.1 (79.2–142.2)	−0.32 (−0.35 to −0.28)	107.3 (78.1–140.0)	112.8 (82.1–145.9)	−0.29 (−0.32 to −0.26)	68.1 (51.1–87.6)	66.1 (49.2–84.8)	−0.16 (−0.18 to −0.15)
High-income North America	97.8 (70.5–128.0)	101.3 (72.3–132.6)	0.11 (0.06 to 0.16)	98.9 (70.8–129.2)	96.3 (69.4–126.0)	0.09 (0.04 to 0.13)	56.3 (41.7–72.5)	54.0 (39.8–69.2)	−0.14 (−0.15 to −0.13)
North Africa and Middle East	44.1 (30.0–59.9)	32.1 (22.1–43.6)	−1.53 (−1.70 to −1.37)	30.8 (21.3–41.8)	42.1 (28.6–57.1)	−1.52 (−1.68 to −1.35)	16.1 (11.6–21.1)	13.5 (9.9–17.8)	−0.84 (−0.93 to −0.76)
Oceania	36.2 (24.2–49.5)	42.7 (28.2–59.9)	0.80 (0.70 to 0.90)	40.9 (27.1–57.3)	34.7 (23.3–47.4)	0.80 (0.71 to 0.90)	12.4 (8.8–16.5)	13.4 (9.1–18.2)	0.38 (0.33 to 0.44)
South Asia	56.4 (37.4–77.5)	60.8 (40.8–83.3)	0.27 (0.24 to 0.30)	58.2 (39.3–79.9)	54.0 (35.8–74.0)	0.27 (0.24 to 0.31)	17.7 (12.7–23.3)	18.6 (13.3–24.6)	0.19 (0.18 to 0.20)
Southeast Asia	31.8 (21.2–43.7)	32.1 (21.6–43.9)	0.20 (0.11 to 0.29)	30.9 (20.9–42.3)	30.4 (20.3–41.8)	0.22 (0.13 to 0.32)	11.5 (8.3–15.3)	11.8 (8.4–15.6)	0.16 (0.12 to 0.20)
Southern Latin America	50.4 (35.0–67.8)	53.1 (37.2–71.3)	0.18 (0.13 to 0.22)	51.2 (36.0–68.8)	48.7 (34.1–65.6)	0.17 (0.12 to 0.22)	24.5 (17.6–31.9)	24.7 (18.0–32.2)	−0.00 (−0.04 to 0.03)
Southern Sub-Saharan Africa	37.9 (25.6–51.3)	43.8 (29.3–59.0)	0.46 (0.12 to 0.79)	42.2 (28.2–57.0)	36.6 (24.7–49.4)	0.45 (0.12 to 0.78)	15.1 (10.9–19.7)	16.3 (11.6–21.4)	0.24 (0.06 to 0.42)
Tropical Latin America	46.6 (32.2–62.2)	38.0 (26.8–50.5)	−0.86 (−0.93 to −0.80)	52.5 (35.3–75.3)	51.1 (35.7–71.0)	−0.86 (−0.92 to −0.79)	19.0 (13.9–24.8)	18.2 (13.5–23.7)	0.46 (0.43 to 0.48)
Western Europe	88.9 (64.5–115.2)	81.8 (59.7–105.8)	−0.33 (−0.38 to −0.27)	94.6 (70.5–123.3)	92.8 (68.0–124.2)	−0.32 (−0.37 to −0.27)	53.3 (39.4–68.7)	50.1 (37.1–64.2)	0.11 (0.09 to 0.14)
Western Sub-Saharan Africa	56.1 (37.4–77.0)	59.6 (39.6–81.5)	0.13 (0.03 to 0.22)	7.2 (4.5–10.3)	7.2 (4.6–10.4)	0.13 (0.04 to 0.22)	17.7 (12.6–23.3)	18.2 (13.0–23.8)	−0.09 (−0.14 to −0.05)

TS, Turner syndrome; ASPR, age-standardized prevalence rate.

UCA had a PR at birth of 838.1 per 100,000, a PR of 674.0 per 100,000 for children younger than 1 year, and an ASPR of 88.4 per 100,000, with its PR still rising in 2019 (Table 3; Supplementary Figure S1). Female PR was higher than male PR in 3 age strata, and the trend of PR for females remained stable, while PR for males was in an upward trend (Table 3; Supplementary Figure S2). PR was trending down in high SDI region, stabilizing in middle-high region, and trending up in other 3 SDI regions (Table 3; Supplementary Figure S1). High-income Asia Pacific and Eastern Europe had the highest PR of UCA in the 3 age strata, with a downward trend (Table 3). There are 5 major regions with rising PR in the 3 age strata, including Andean Latin America (highest), Oceania, South Asia, Southern Latin America, and Southern Sub-Saharan Africa.

**Table 3:** Prevalence rate (PR) for urogenital congenital anomalies in 1990 and 2019 and their estimated annual percent change (EAPCs).

UCA (per 100,000)	PR at birth			PR for children younger than 1 year			ASPR		
	1990 (95% UI)	2019 (95% UI)	EAPC (1990–2019, 95%CI)	1990 (95% UI)	2019 (95% UI)	EAPC (1990–2019, 95%CI)	1990 (95% UI)	2019 (95% UI)	EAPC (1990–2019, 95%CI)
Global	805.3 (565.9–1147.9)	838.1 (587.7–1198.6)	0.13 (0.06 to 0.21)	650.6 (465.7–907.7)	674.0 (481.9–939.9)	0.11 (0.04 to 0.18)	89.2 (70.5–109.7)	88.4 (69.9–108.7)	0.05 (0.02 to 0.08)
**Sex**									
Female	835.2 (580.0–1175.8)	855.3 (600.5–1209.8)	0.06 (−0.02 to 0.14)	674.5 (479.7–921.3)	690.2 (496.1–956.1)	0.04 (−0.03 to 0.12)	92.4 (73.4–113.4)	91.9 (73.1–113.6)	0.01 (−0.01 to 0.03)
Male	777.3 (544.8–1113.2)	822.1 (577.6–1189.2)	0.20 (0.13 to 0.27)	628.1 (448.0–887.0)	658.8 (466.6–927.6)	0.18 (0.11 to 0.24)	86.1 (67.4–106.3)	85.1 (66.8–105.2)	0.10 (0.05 to 0.14)
**SDI region**									
Low	857.7 (591.2–1246.1)	860.6 (598.0–1248.4)	0.07 (0.00 to 0.13)	690.5 (480.3–987.7)	690.7 (491.4–977.6)	0.07 (0.00 to 0.13)	88.9 (69.9–110.1)	87.0 (68.0–108.2)	0.12 (0.08 to 0.17)
Low-middle	866.5 (598.8–1252.3)	907.1 (634.2–1315.3)	0.23 (0.17 to 0.30)	697.1 (491.7–983.7)	729.1 (517.7–1029.3)	0.21 (0.15 to 0.28)	94.5 (74.8–116.9)	90.0 (70.7–111.9)	0.17 (0.13 to 0.21)
Middle	740.6 (521.1–1051.0)	793.7 (554.6–1115.3)	0.18 (0.07 to 0.28)	599.1 (428.2–830.1)	639.4 (458.9–885.5)	0.16 (0.05 to 0.26)	85.0 (67.0–104.9)	81.2 (64.3–99.5)	0.13 (0.08 to 0.18)
Middle-high	765.1 (540.8–1089.6)	811.5 (572.8–1144.2)	0.13 (−0.03 to 0.28)	626.0 (452.8–869.3)	652.9 (469.3–893.5)	0.08 (−0.05 to 0.21)	87.7 (69.3–107.9)	92.6 (73.8–114.5)	−0.04 (−0.20 to 0.11)
High	811.3 (595.8–1096.7)	714.4 (525.1–959.4)	−0.64 (−0.78 to −0.51)	661.5 (490.8–888.2)	583.0 (437.0–775.6)	−0.65 (−0.79 to −0.52)	86.3 (69.9–104.3)	96.2 (77.0–116.9)	−0.50 (−0.58 to −0.41)
**GBD region**									
Andean Latin America	547.4 (389.7–774.9)	625.3 (454.9–881.5)	0.50 (0.43 to 0.57)	436.9 (313.8–597.7)	493.4 (363.0–676.6)	0.48 (0.43 to 0.54)	62.9 (50.5–76.7)	57.8 (46.3–70.2)	0.36 (0.33 to 0.39)
Australasia	955.2 (677.4–1338.4)	795.3 (567.2–1099.8)	−0.69 (−0.74 to −0.65)	728.0 (534.7–991.3)	630.9 (457.6–856.8)	−0.54 (−0.57 to −0.52)	81.4 (64.8–100.7)	83.6 (66.8–103.3)	−0.12 (−0.13 to −0.10)
Caribbean	596.0 (423.9–824.1)	603.1 (428.9–842.5)	−0.14 (−0.22 to −0.07)	481.5 (346.8–648.0)	492.9 (351.6–676.0)	−0.07 (−0.14 to −0.00)	69.7 (55.3–87.1)	67.1 (53.9–82.8)	0.13 (0.09 to 0.16)
Central Asia	794.7 (548.2–1126.9)	826.9 (568.6–1173.1)	−0.06 (−0.17 to 0.05)	640.9 (448.2–892.5)	668.4 (468.1–921.3)	−0.04 (−0.14 to 0.06)	88.6 (70.3–110.5)	85.4 (67.3–106.9)	0.06 (0.02 to 0.09)
Central Europe	857.9 (596.6–1258.3)	749.0 (522.7–1104.3)	−0.45 (−0.67 to −0.22)	645.9 (463.0–904.0)	593.0 (423.4–816.8)	−0.37 (−0.50 to −0.24)	83.1 (65.6–103.8)	85.2 (67.7–105.4)	−0.08 (−0.11 to −0.05)
Central Latin America	857.0 (605.1–1217.1)	831.6 (579.3–1176.2)	0.13 (−0.18 to 0.43)	689.9 (486.9–971.6)	673.2 (477.2–938.4)	0.08 (−0.21 to 0.37)	95.9 (76.4–118.9)	95.6 (76.0–118.5)	−0.08 (−0.29 to 0.12)
Central Sub-Saharan Africa	910.4 (628.2–1303.0)	923.4 (622.4–1346.5)	−0.17 (−0.31 to −0.03)	732.2 (511.8–1032.2)	731.1 (514.5–1032.8)	−0.13 (−0.23 to −0.03)	91.3 (71.8–112.3)	90.2 (70.4–112.2)	0.11 (0.08 to 0.14)
East Asia	519.8 (360.8–723.0)	617.9 (428.2–864.7)	−0.01 (−0.56 to 0.54)	436.1 (307.9–590.3)	498.3 (349.7–679.7)	−0.09 (−0.58 to 0.41)	68.3 (53.7–83.8)	73.7 (58.7–91.9)	−0.12 (−0.22 to −0.02)
Eastern Europe	1159.1 (799.5–1679.0)	1115.6 (770.4–1600.4)	−0.17 (−0.28 to −0.05)	941.1 (668.8–1332.8)	910.5 (642.5–1275.4)	−0.16 (−0.26 to −0.06)	120.6 (95.1–151.0)	121.6 (95.9–150.8)	−0.16 (−0.21 to −0.11)
Eastern Sub-Saharan Africa	871.5 (597.1–1258.2)	812.9 (566.1–1171.7)	−0.07 (−0.15 to 0.01)	693.1 (480.7–981.0)	656.2 (467.3–932.2)	−0.04 (−0.11 to 0.03)	84.5 (66.3–103.9)	83.4 (65.2–103.0)	0.09 (0.03 to 0.15)
High-income Asia Pacific	1309.7 (926.7–1862.8)	1014.7 (724.5–1398.3)	−1.22 (−1.44 to −1.01)	1084.5 (777.5–1516.6)	854.7 (627.8–1160.1)	−1.08 (−1.26 to −0.89)	132.0 (105.2–162.1)	147.3 (115.7–183.0)	−0.39 (−0.45 to −0.33)
High-income North America	597.0 (438.4–804.3)	550.0 (409.0–729.5)	−0.48 (−0.59 to −0.37)	489.6 (362.5–650.9)	441.2 (335.4–570.2)	−0.66 (−0.81 to −0.52)	67.0 (55.0–80.3)	78.1 (63.0–95.3)	−0.86 (−1.05 to −0.66)
North Africa and Middle East	987.8 (690.1–1467.4)	911.9 (635.3–1342.7)	−0.35 (−0.48 to −0.23)	796.3 (560.9–1155.0)	740.7 (528.1–1077.5)	−0.32 (−0.44 to −0.21)	106.6 (83.4–132.2)	107.5 (83.9–134.5)	−0.10 (−0.16 to −0.04)
Oceania	653.7 (453.8–910.5)	673.2 (460.8–962.8)	0.24 (0.11 to 0.38)	528.7 (373.1–719.9)	542.5 (383.6–754.6)	0.17 (0.08 to 0.26)	77.4 (61.1–96.3)	74.6 (59.4–92.7)	0.07 (0.04 to 0.09)
South Asia	973.1 (667.2–1421.6)	1044.1 (719.2–1528.8)	0.35 (0.26 to 0.43)	786.1 (553.6–1118.9)	836.4 (591.9–1189.1)	0.31 (0.23 to 0.39)	105.1 (82.0–130.8)	99.9 (77.4–125.4)	0.19 (0.15 to 0.23)
Southeast Asia	612.9 (418.9–874.1)	608.0 (412.6–876.5)	−0.15 (−0.20 to −0.09)	494.6 (346.9–686.8)	493.0 (342.9–681.1)	−0.11 (−0.16 to −0.07)	67.2 (53.7–84.5)	66.2 (52.9–82.7)	0.04 (0.02 to 0.05)
Southern Latin America	747.4 (551.5–1059.7)	783.1 (561.7–1108.8)	0.30 (0.23 to 0.36)	593.1 (442.8–810.7)	623.5 (459.5–849.8)	0.28 (0.23 to 0.33)	84.0 (67.4–102.9)	80.0 (63.8–97.8)	0.19 (0.13 to 0.24)
Southern Sub-Saharan Africa	830.1 (572.0–1169.8)	888.3 (617.2–1241.6)	0.47 (0.35 to 0.60)	670.4 (480.1–908.7)	715.3 (506.3–974.9)	0.44 (0.32 to 0.56)	91.5 (72.6–113.6)	85.9 (67.7–106.4)	0.28 (0.20 to 0.35)
Tropical Latin America	594.2 (427.4–839.5)	482.3 (350.5–672.3)	−0.81 (−1.01 to −0.61)	469.4 (344.9–651.9)	382.3 (282.0–516.2)	−0.81 (−1.00 to −0.62)	54.6 (43.9–66.3)	64.8 (51.3–79.5)	−0.74 (−0.86 to −0.62)
Western Europe	728.2 (555.0–984.8)	726.1 (539.5–972.3)	0.10 (−0.01 to 0.20)	592.1 (454.8–779.4)	587.7 (444.4–781.1)	0.04 (−0.06 to 0.14)	80.8 (65.1–97.9)	82.0 (66.3–98.7)	−0.13 (−0.20 to −0.07)
Western Sub-Saharan Africa	899.8 (624.6–1279.5)	884.4 (613.2–1265.7)	0.02 (−0.05 to 0.09)	715.9 (506.1–998.8)	713.1 (512.0–1007.4)	0.04 (−0.02 to 0.11)	90.0 (70.9–111.4)	86.2 (67.7–107.0)	0.16 (0.12 to 0.20)

UCA, urogenital congenital anomalies; ASPR, age-standardized prevalence rate.

### Global DALYs trends of RCBDs from 1990 to 2019

**Figure 1:**
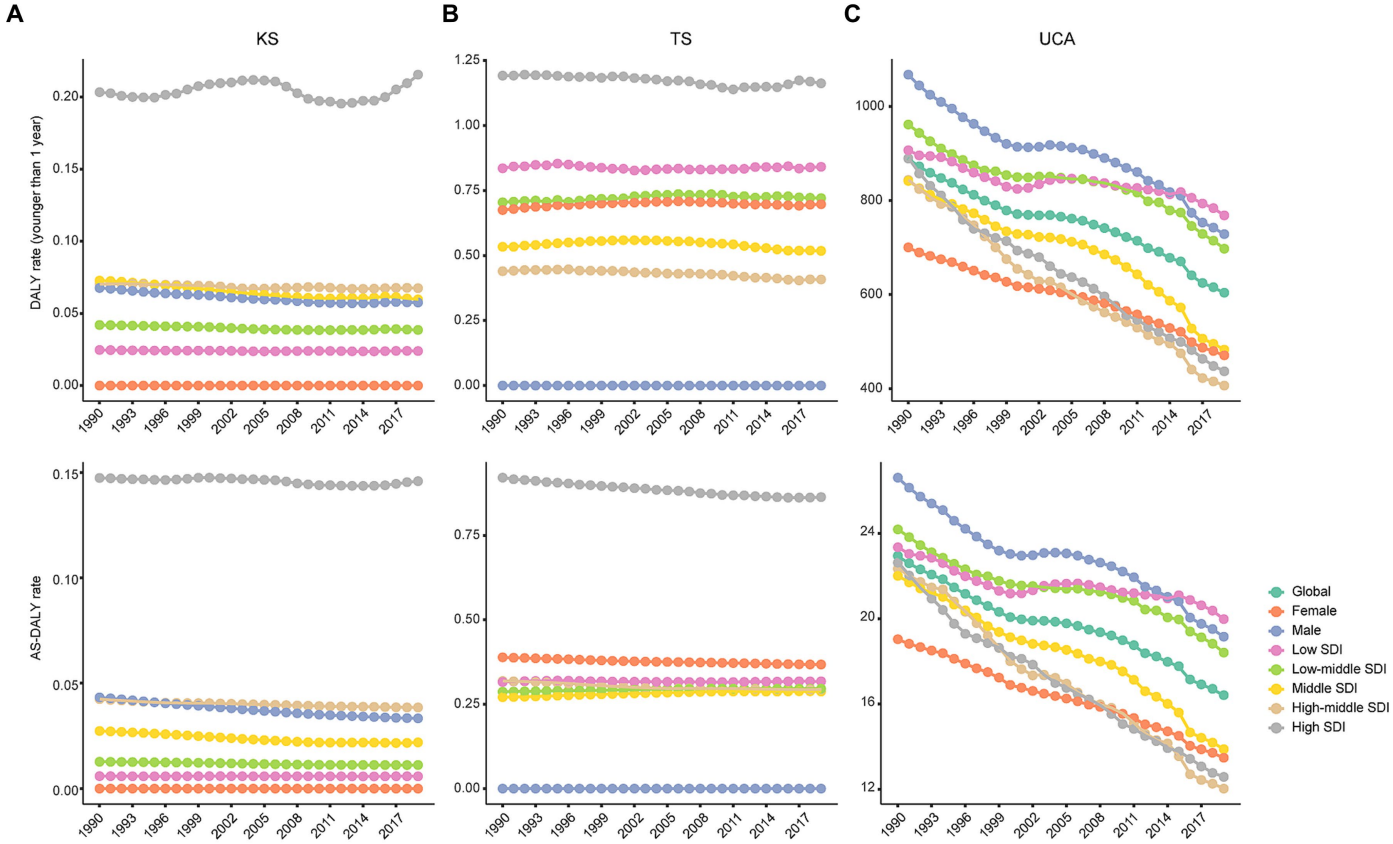
Trends of global DALY rates by gender and SDIs from 1990 to 2019. KS, Klinefelter syndrome **(A)**; TS, Turner syndrome **(B)**; UCA, urogenital congenital anomalies **(C)**; DALY, disability-adjusted life years; SDI, sociodemographic index.

**Figure 2:**
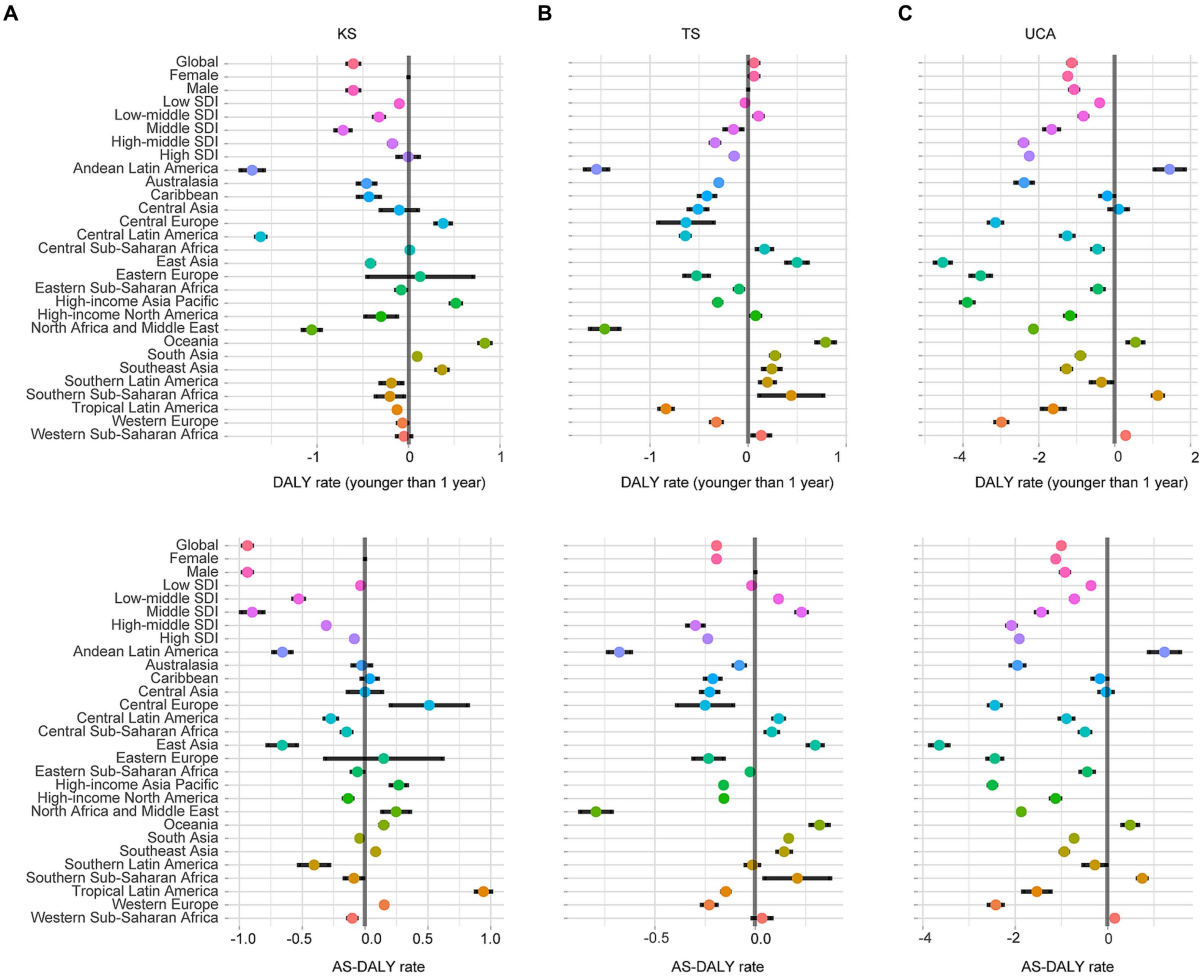
EAPCs of DALY rates at the regional levels. KS, Klinefelter syndrome **(A)**; TS, Turner syndrome **(B)**; UCA, urogenital congenital anomalies **(C)**; DALY, disability-adjusted life years; SDI, sociodemographic index.

Globally, the DALY rates for KS and TS were relatively low, less than 1 per 100,000 (Figure 1). For KS and TS, the DALY rates for children younger than 1 year and the AS-DALY rates were highest in high SDI region. The DALY rates for children younger than 1 year and AS-DALY rates of KS showed a downward trend globally and across the 5 SDI regions (Figure 2). The DALY rates for children younger than 1 year in KS increased in 5 regions, and the AS-DALY rates of KS increased in 7 regions, both of which include Central Europe, High-income Asia Pacific, Oceania, Southeast Asia (Figure 2). The DALY rates (children younger than 1 year and age-standardized) for TS increased in Central Sub-Saharan Africa, East Asia, Oceania, South Asia, Southeast Asia, and Southern Sub-Saharan Africa (Figure 2).

The global DALY rate of UCA for children younger than 1 year was 603.9 per 100,000 (95% UI, 440.3–804.5), global AS-DALY rate of UCA was 16.4 per 100,000 (95%UI, 12.4–21.0) in 2019 (Figure 1). The DALY rates (children younger than 1 year and age-standardized) in women with UCA were lower than in men (e.g., AS-DALYs in 2019: female, 12.5 per 100,1,000; male, 19.2 per 100,000) (Figure 1). In 2019, the DALY rates of UCA (children younger than 1 year and age-standardized) were higher in the low SDI and low-middle SDI regions, while fell to lower levels in the other 3 SDI regions (Figure 1). The DALY rates of UCA continued to decline globally and in the 5 SDI regions (Figures 1, 2). Among the 21 GBD regions, Andean Latin America, Oceania, Southern Sub-Saharan Africa and Western Sub-Saharan Africa continued to show an increasing trend in DALY rates of UCA (children younger than 1 year and age-standardized) (Figure 2).

### National DALYs trends of RCBDs from 1990 to 2019

**Figure 3:**
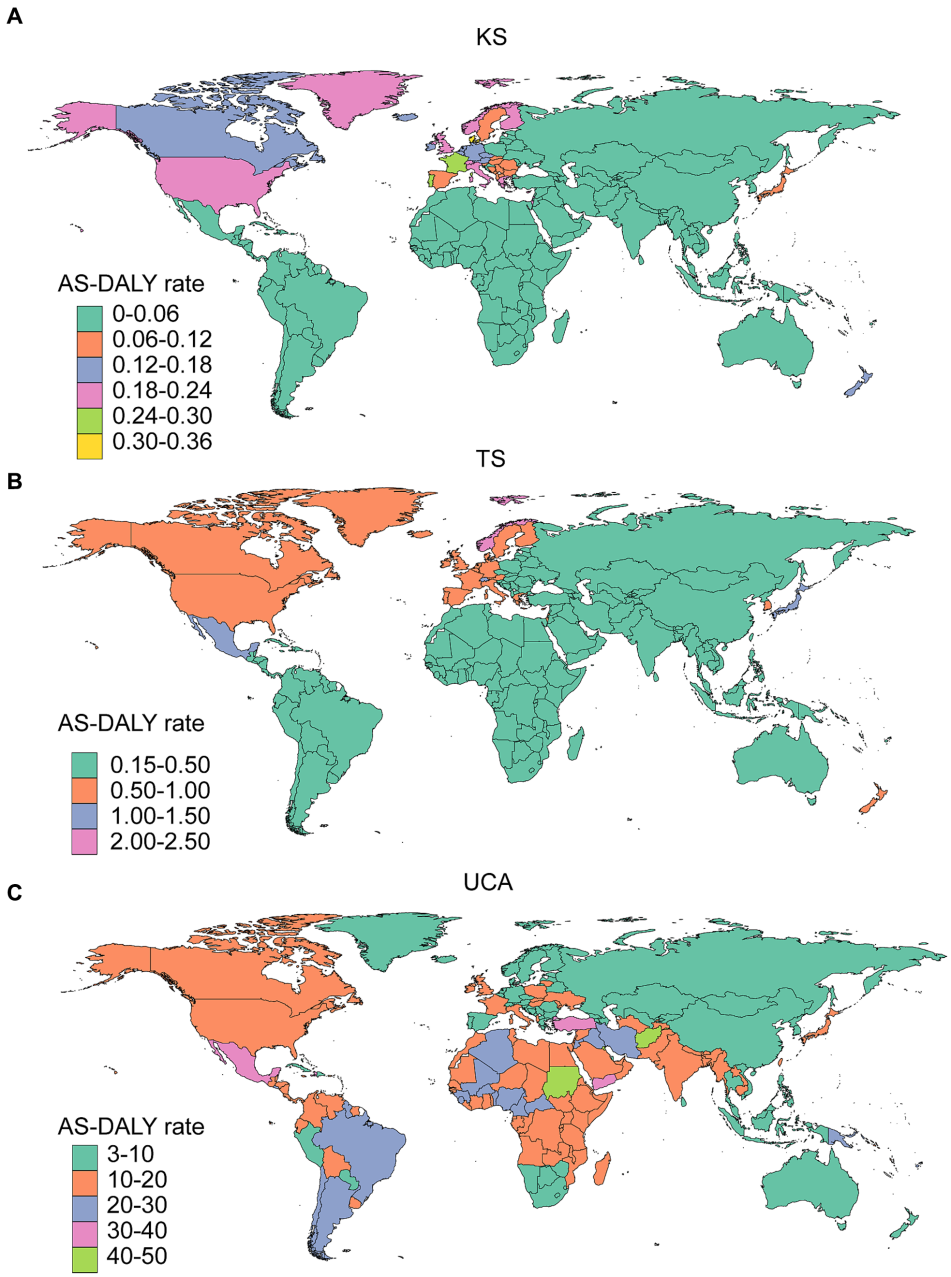
Global trends in the AS-DALY rates in 204 countries and territories in 2019. KS, Klinefelter syndrome **(A)**; TS, Turner syndrome **(B)**; UCA, urogenital congenital anomalies **(C)**.

Results from this study indicated that in 2019, the top 4 countries with AS-DALY rates in KS were Denmark, Portugal, France, and Malta, the top 4 countries with AS-DALY rates in TS were Norway, Japan, Mexico, and Switzerland, and the top 4 countries with AS-DALY rates in UCA were Afghanistan, Sudan, Kuwait, and Yemen (Figure 3; Supplementary Table S1). There was a high degree of consistency in the distribution of AS-DALY rates in KS and TS, with countries in the South America, Asia, Africa, and Australia regions having relatively low AS-DALY rates (Figure 3; Supplementary Table S1). The AS-DALY rates in UCA were higher in North America, South America, and Africa (Figure 3; Supplementary Table S1). RCBDs seemed to have a regional character, with rates of AS-DALYs in KS and TS being higher mainly in high-income economies, while in UCA being higher in both high-income and low-income economies. The national distribution of DALY rates for children younger than 1 year was characterized in Supplementary Figure S3.

**Figure 4:**
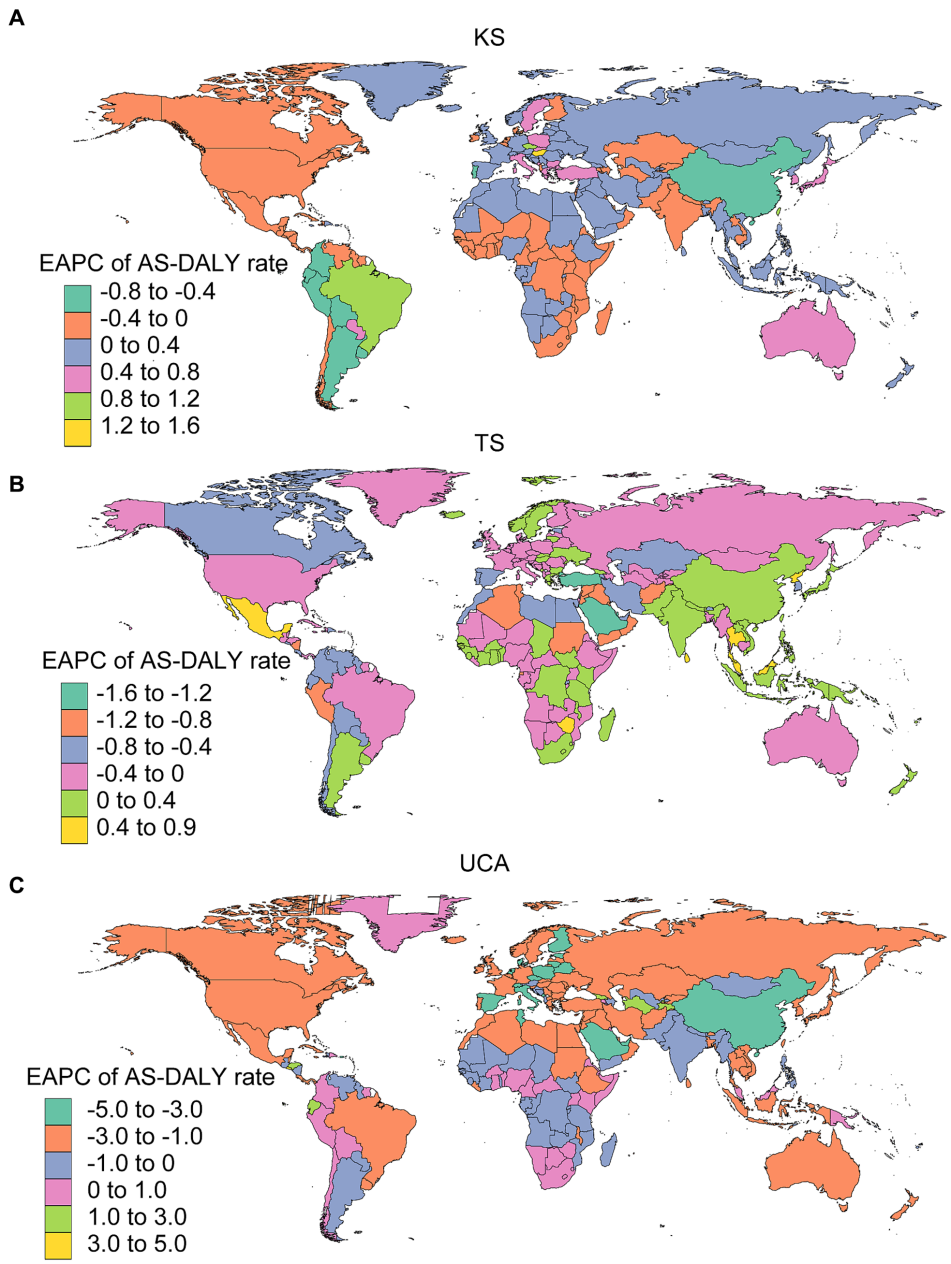
EAPCs of AS-DALY rates in 204 countries and territories. KS, Klinefelter syndrome **(A)**; TS, Turner syndrome **(B)**; UCA, urogenital congenital anomalies **(C)**.

In KS, there were 87 countries and territories with decreasing AS-DALY rates, and the top 3 were Hungary, Federative Republic of Brazil, and Czech Republic; in TS, there were 57 countries and territories with decreasing AS-DALY rates, and the top 3 were Democratic People’s Republic of Korea, Zimbabwe, and Niue; in UCA, there were 33 countries and territories with decreasing AS-DALY rates, and the top 3 were El Salvador, Tajikistan, and Ecuador (Figure 4; Supplementary Table S1). As with the trend in AS-DALY rates of KS, TS and UCA, there was no clear regional selectivity in the rates of DALYs for children younger than 1 year (Supplementary Figure S4).

### Correlations of EAPCs of DALY rates with SDI

**Figure 5:**
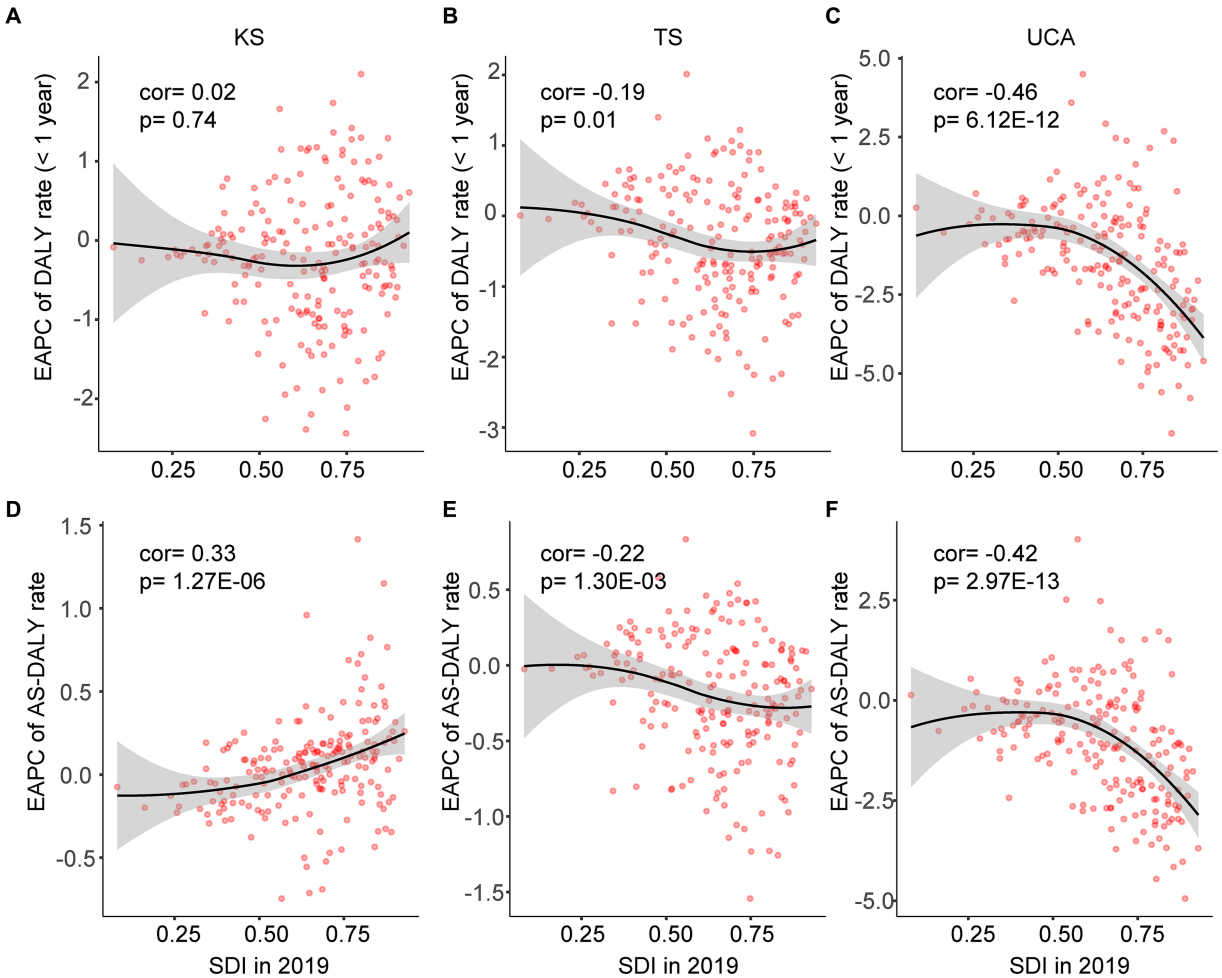
Pearson correlation was analyzed between EAPCs of DALY rates from 1990 to 2019 and SDI in 2019 at the country and territorial levels. Cor, correlation coefficient. KS, Klinefelter syndrome **(A,D)**; TS, Turner syndrome **(B,E)**; UCA, urogenital congenital anomalies **(C,F)**.

There was a significant negative correlation between the EAPCs of DALY rates (children younger than 1 year) in TS and UCA and the SDI (in 2019) (Figures 5B,C). Figures 5E,F also showed similar results for EAPCs of AS-DALY rates for TS and UCA with SDI (in 2019). The DALY rates in TS and UCA declined faster in countries with higher SDI. The EAPCs for AS-DALY rates in KS were positively correlated with the SDI, with countries with higher SDI showing an increasing trend in AS-DALY rates (Figure 5D). The EAPCs of PR at birth, PR for children younger than 1 year and ASPR also were associated with SDI, all 3 age groups in TS and UCA were negatively associated with SDI, and EAPCs for ASPR in KS were positively associated with SDI (Supplementary Figure S5).

## Discussion

In this study, temporal trends in the prevalence and DALY rates of RCBDs were comprehensively analyzed at the global, regional, and national level. Overall, PR at birth, PR for children under 1 year, and ASPR were decreasing in KS; PR at birth and PR for children under 1 year in TS and UCA were increasing, and ASPRs declined in TS and increased in UCA. Globally, DALY rates for children younger than 1 year were on a downward trend in KS and UCA, while they were still rising for TS. And AS-DALY rates were all on a downward trend in KS, TS, and UCA. The DALY rates of KS, TS, and UCA was higher in high-income countries, although their DALY rates were on a downward trend. In addition, the burdens of TS and UCA went down with increasing SDI, whereas the burden of KS increased with increasing SDI.

The PR of KS has been reported to be 0.1–0.2% in the general population and up to 3.1% in the infertile male population (6, 11–13). These prevalences were undoubtedly higher than the prevalence in the present study. The main reason may be that the cases in the GBD study excluded situations such as all terminations of pregnancy following prenatal diagnosis, resulting in a low prevalence. In addition, the current PR is generally considered to be lower than the actual value, with two-thirds of cases of the syndrome undetected (6). For example, patients with chromosomally mosaic KS usually showed few clinical symptoms and led almost normal lives (6).

The analysis further suggested that the global PR of KS (three age strata) and DALY rates (two age strata) declined from 1990 to 2019, and those of 5 SDI regions were also on a decreasing or stabilizing trend. Within this overall trend, individual countries and territories have shown an upward trend in PR and DALY rates. This indicated that healthcare in KS have improved globally. In addition, trends in ASPR and AS-DALY rates were positively correlated with SDI. This is well understood; countries with higher levels of social development are better able to detect undetected KS in the population, and an increase in the number of KS cases is followed by an increase in DALY rates.

The PR of TS is consistent with previously reported data, approximately 50 per 100,000 in live-born girls (8, 10). The majority of all embryos or fetuses with TS are spontaneously aborted in the first and second trimesters of pregnancy (9, 14). Live-born girls with TS are usually diagnosed on the basis of ultrasound-detected hydrops fetalis findings, abnormal levels of human chorionic gonadotropin, unconjugated estriol, and alpha-fetoprotein detected by maternal serum screening, and abnormal fetal karyotype findings due to advanced maternal age. Or in adolescence because they did not reach puberty, and in adulthood due to repeated pregnancy loss, most patients with TS are diagnosed (10).

The global ASPR and AS-DALY rates for TS were decreasing, whereas PR at birth, and PR and DALY rates for children less than 1 year were still increasing. On the one hand, the global prevalence of TS is generally decreasing in all age groups. On the other hand, improved medical care may lead to improved survival and early diagnosis of patients with TS. At the level of 5 SDI regions, the trends of PRs and DALY rates were characterized by “low at both ends and high in the middle,” i.e., the PRs and DALY rates in the low and high SDI regions were on a downward trend, while those in the middle-low and/or middle SDI regions were on an upward trend. The countries with higher ASPRs for TS were concentrated in North America and Europe. This is in line with the previous statement that better medical care is more likely to detect TS. Additionally, countries with higher SDI generally exhibited a downward trend in the PRs and DALY rates for TS.

UCA includes a variety of birth defects, such as polycystic kidney and double ureter, and there is a lack of data on detailed entries for analysis. This study used GBD study to illustrate the global characteristics of UCA. The trend in PRs (three age strata) for global UCA was increasing, while the trend in DALY rates (two age strata) was decreasing. This study found a higher PR in women than in men, with a trend that has remained stable over 30 years (the trend for PR in men was upward), and a lower rate of DALYs in women than in men, with a downward trend in both. It is not clear why PR is higher and DALY rate lower in women; it may be that men generally do not experience UCA, and when they do, the harm is greater. The trend in PRs (3 age strata) was downward only in the high SDI region. Early prenatal diagnosis of fatal or significant-harm UCA may have contributed to its declining PR. The decline in the DALY rates in all SDI regions suggested an improvement of health in the patients with UCA, despite the unavailability of high-quality prenatal diagnosis in some areas or other reasons. Countries with high DALY rates in UCA were mainly in South America and Africa. The relationship between SDI and trends in PR and DALY rates also suggested that the higher the SDI, the more pronounced the downward trend.

Our strengths were that this study presented the latest epidemiological patterns of the burdens of reproduction-related congenital defects at global, regional, and national levels across age, sex, and SDI categories. This study also investigated the correlation between SDI and the burdens of reproduction-related congenital defects. In addition, this study described, for the first time, long-term trends in the PR for the burdens of RCBDs in 204 countries and territories from 1990 to 2019. The estimates from the GBD study were derived using reliable data collection and analysis methods. However, there might be some potential biases in the data that limit the accuracy of the interpretation of the results. Secondly, the GBD study lacked data on segmented diseases in UCA, which prevented further analysis of trends. Finally, this study only addressed RCBDs; more types of birth defects were not analyzed in this manuscript.

## Conclusion

The population-level impact of RCBD shows in years lost to disability/disease and a reduced life expectancy *per capita*. The global burdens of RCBDs have decreased since 1990. ASPR in UCA was still on the rise and UCA requires further public health attention due to high disability rates. In addition, the DALY rates of KS was increasing more rapidly in more developed regions, and it is recommended that early screening for KS should be improved to treat it earlier and reduce adverse outcomes. This study has brought about important advances in global population health, especially child health. The findings can help public health policymakers implement cost-effective interventions to reduce the burdens of RCBDs.

## Data availability statement

All data are available from publicly available databases (https://ghdx.healthdata.org/gbd-2019; data access, August 14, 2023). The original contributions presented in the study are included in the article/Supplementary material, further inquiries can be directed to the corresponding author.

## Ethics statement

This study used publicly available data, contained no personal or medical information about identifiable living individuals, and did not involve animal subject.

## Author contributions

LS: Data curation, Investigation, Methodology, Writing – original draft. JL: Data curation, Investigation, Methodology, Writing – review & editing. HZ: Conceptualization, Writing – review & editing. YZ: Conceptualization, Writing – review & editing.

## Footnotes

^1^http://ghdx.healthdata.org/gbd-results-tool
