# Supplementary material for: Global, regional and national burdens of reproduction-related congenital birth defects, 1990–2019
Source: Front Public Health. 2024 Feb 26;12:1328282. doi: 10.3389/fpubh.2024.1328282 (PMC10925714; doi:10.3389/fpubh.2024.1328282)
Supplement: Supplementary file 1 [file Data_Sheet_1.docx]

**Supplementary Methods**

There are three main standardized tools: Cause of Death Ensemble model (CODEm), spatiotemporal Gaussian process regression (ST-GPR), and DisMod-MR. Previous publications and the appendix provide more details on these general GBD methods(1). Briefly, CODEm is a highly systematized tool to analyze cause of death data using an ensemble of different modelling methods for rates or cause fractions with varying choices of covariates that perform best with out-of-sample predictive validity testing. DisMod-MR is a Bayesian meta-regression tool that allows evaluation of all available data on incidence, prev­alence, remission, and mortality for a disease, enforcing consistency between epidemiological para­ meters. ST-GPR is a set of regression methods that borrow strength between locations and over time for single metrics of interest, such as risk factor exposure or mortality rates.

Disability-adjusted life-year (DALY) were calculated by summing years of life lost (YLLs) due to premature mortality and years of life lived with disability (YLDs), thereby incorporating both fatal and non-fatal burden. Since Klinefelter syndrome and Turner syndrome were estimated as causing non-fatal disease burden only, DALYs estimates were obtained from YLDs. And DALYs for urogenital congenital anomalies were obtained from YLLs and YLDs. YLLs are estimated as the multiplication of counts of death and a standard, “ideal,” remaining life expectancy at the age of death. The standard life expectancy is derived from the lowest observed mortality rates in any population in the world greater than 5 million. YLDs are estimated as the product of prevalence of individual consequences of disease (or “sequelae”) times a disability weight that quantifies the relative severity of a sequela as a number between zero (representing “full health”) and 1 (representing death). Disability weights have been estimated in nine population surveys and an open-access internet survey in which respondents are asked to choose the “healthier” between random pairs of health states that are presented with a short description of the main features(2).

Covariates having a specified positive correlation with congenital birth defects mortality were maternal alcohol consumption, proportion of live births in women aged 35+, age-standardized diabetes prevalence, indoor air pollution, and reproductive age-standardized smoking prevalence(2). Covariates having a specified negative correlation with congenital birth defects mortality were measles vaccine coverage, education (years per capita), and legality of abortion(2).

All parameter estimates generated in the GBD 2019 were accompanied by 95% uncertainty intervals (UIs). The 95% uncertainty interval is reported as the 25th and 975th values of the distribution.

Reference

1. Global burden of 369 diseases and injuries in 204 countries and territories, 1990-2019: a systematic analysis for the Global Burden of Disease Study 2019. Lancet. 2020;396(10258):1204-22.

2. Global, regional, and national burden of congenital heart disease, 1990-2017: a systematic analysis for the Global Burden of Disease Study 2017. Lancet Child Adolesc Health. 2020;4(3):185-200.

**Supplementary Figure 1.** Estimated annual percentage changes (EAPCs) of prevalence at the regional levels. PR, prevalence rate; ASPR, age-standardized PR; SDI, sociodemographic index.

**
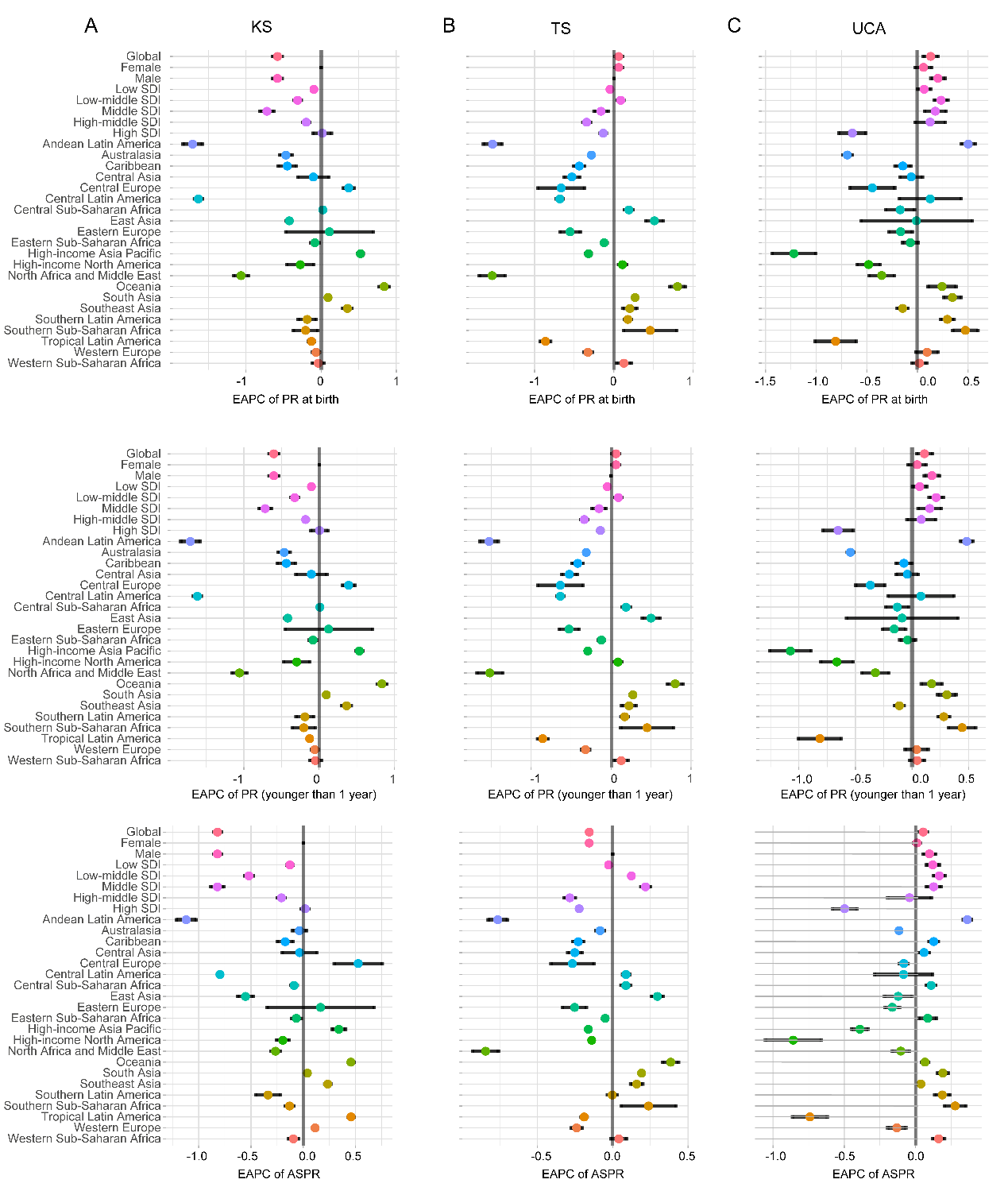
**

**Supplementary Figure 2.** Trends of global prevalence by gender and SDIs from 1990 to 2019. PR, prevalence rate; ASPR, age-standardized PR; SDI, sociodemographic index. The KS for males overlaps the global level and the TS for females overlaps the global level.


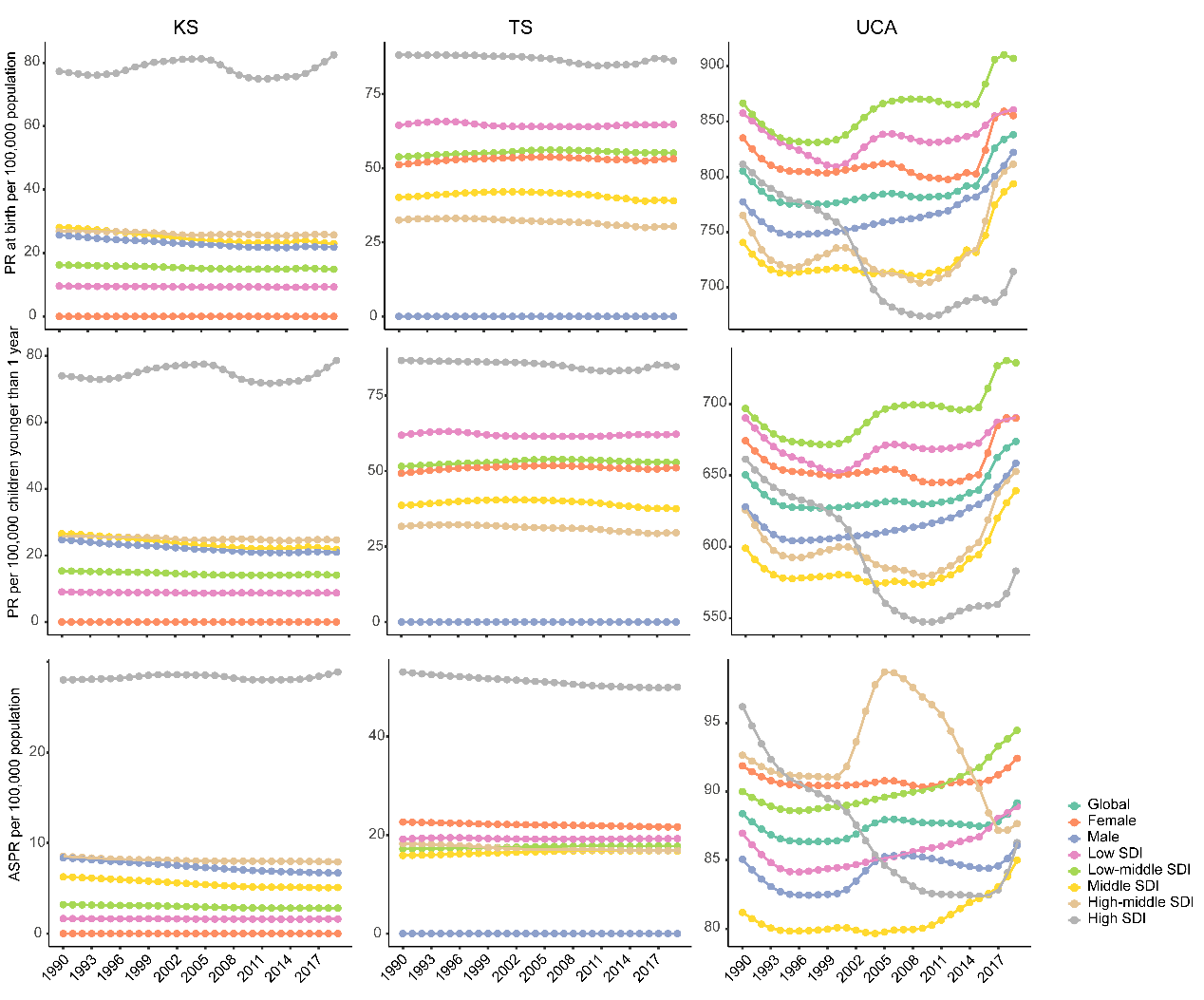


**Supplementary Figure 3.** Global trends in the DALY rate (children younger than 1 year) in 204 countries and territories in 2019.

**
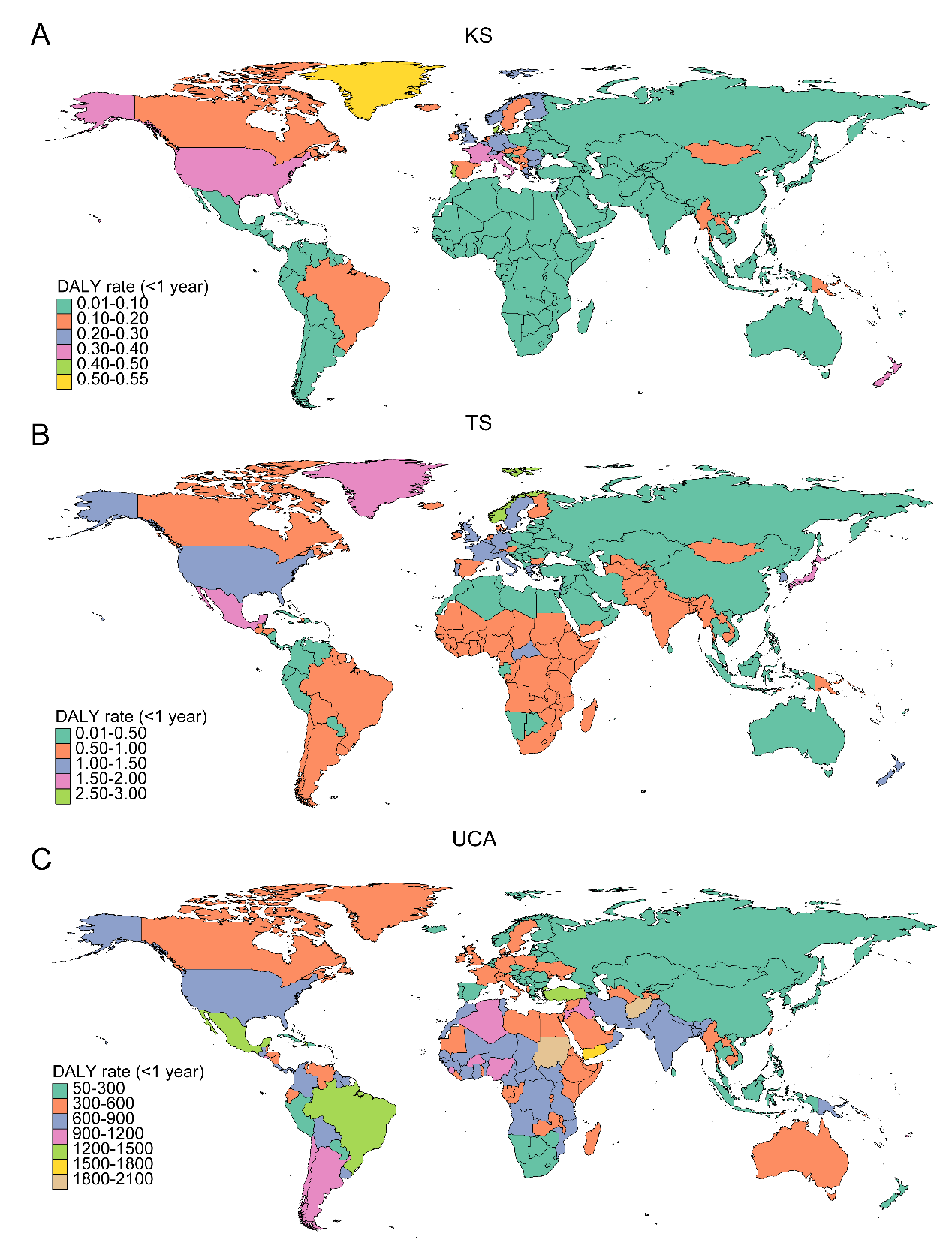
**

**Supplementary Figure 4.** Global trends in the EAPCs of DALY rate (children younger than 1 year) in 204 countries and territories.


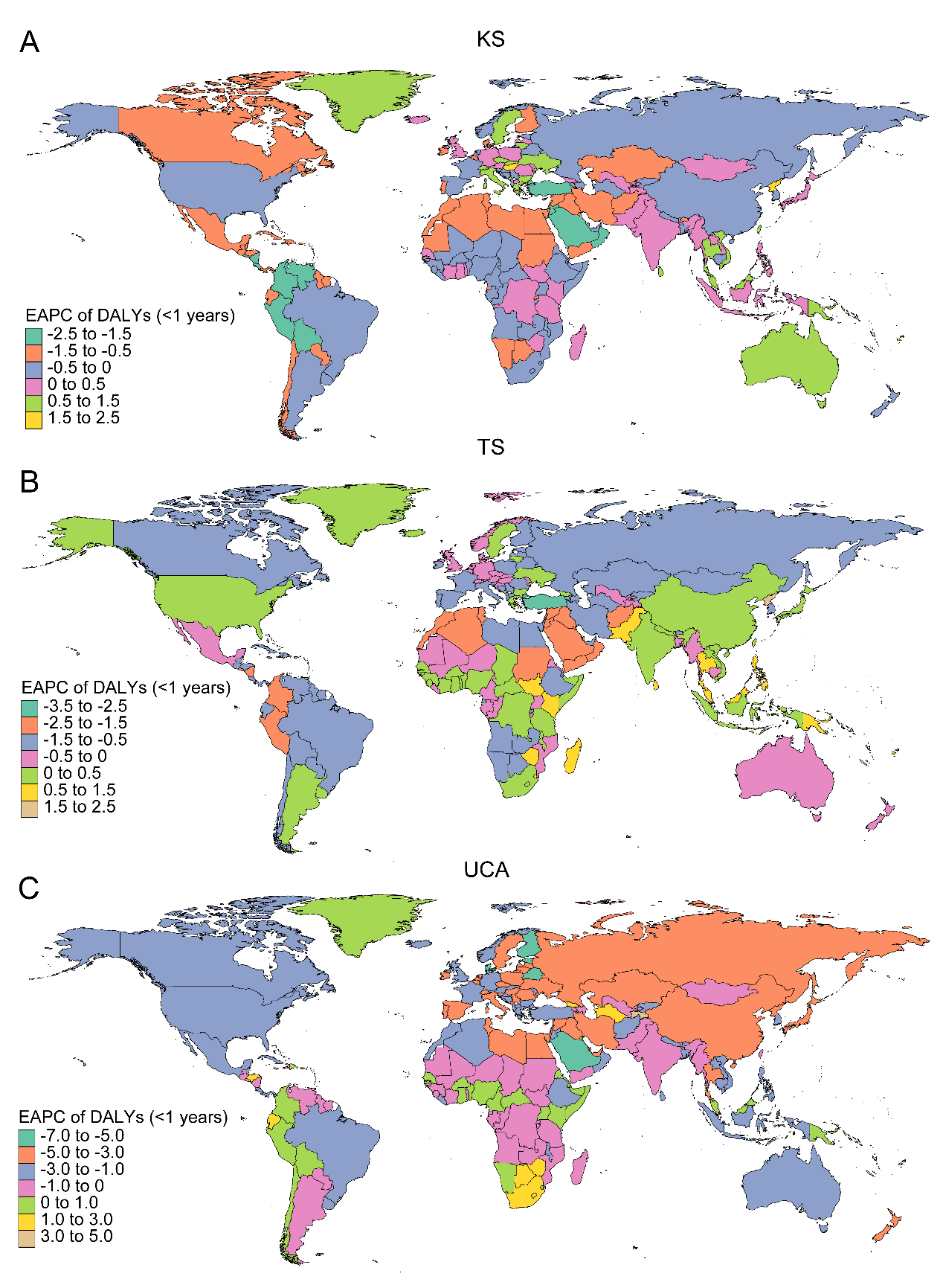


**Supplementary Figure 5.** Pearson correlation was analyzed between EAPCs of PRs and DALY rate from 1990 to 2019 and SDI in 2019 at the country and territorial levels. cor, Correlation coefficient.


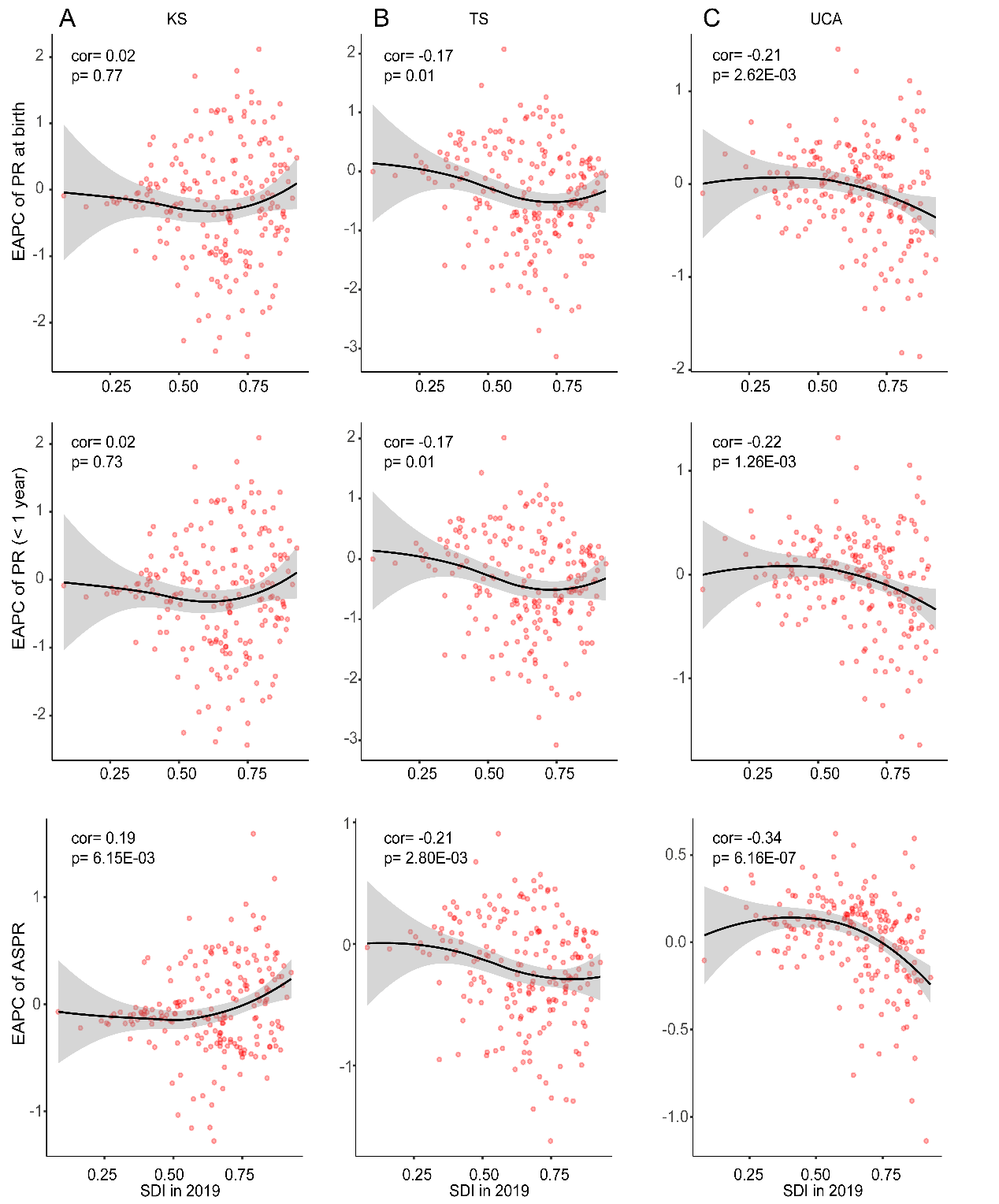


**Supplementary Table.** National EAPCs of AS-DALY rate of reproductive-related congenital birth defects from 1990 to 2019.

|  | location | cause | EAPC | UCI | LCI |
| --- | --- | --- | --- | --- | --- |
| 1 | Luxembourg | UCA | -1.79 | -1.47 | -2.11 |
| 2 | Belgium | UCA | -2.97 | -2.63 | -3.30 |
| 3 | Saint Helena | UCA | -0.30 | -0.20 | -0.39 |
| 4 | Chile | UCA | 0.04 | 0.44 | -0.36 |
| 5 | Uruguay | UCA | -2.50 | -2.34 | -2.66 |
| 6 | Saint Kitts and Nevis | UCA | -0.24 | -0.08 | -0.41 |
| 7 | Cook Islands | UCA | -2.22 | -2.14 | -2.30 |
| 8 | Dominica | UCA | 0.98 | 1.03 | 0.92 |
| 9 | Senegal | UCA | -0.05 | 0.11 | -0.20 |
| 10 | San Marino | UCA | -1.45 | -1.32 | -1.58 |
| 11 | Bolivia (Plurinational State of) | UCA | 0.53 | 0.83 | 0.23 |
| 12 | Dominican Republic | UCA | 0.31 | 0.75 | -0.13 |
| 13 | Greenland | UCA | 0.19 | 0.85 | -0.47 |
| 14 | Guam | UCA | 1.72 | 2.10 | 1.33 |
| 15 | Tokelau | UCA | -1.38 | -1.24 | -1.51 |
| 16 | Sierra Leone | UCA | -0.71 | -0.58 | -0.83 |
| 17 | Philippines | UCA | -0.62 | -0.56 | -0.67 |
| 18 | Ecuador | UCA | 2.48 | 2.97 | 1.99 |
| 19 | Panama | UCA | -1.98 | -1.73 | -2.23 |
| 20 | Samoa | UCA | -1.07 | -0.95 | -1.19 |
| 21 | Sri Lanka | UCA | -1.19 | -0.96 | -1.42 |
| 22 | Venezuela (Bolivarian Republic of) | UCA | -0.05 | 0.14 | -0.24 |
| 23 | Peru | UCA | 0.69 | 1.13 | 0.26 |
| 24 | Thailand | UCA | -2.11 | -1.99 | -2.22 |
| 25 | China | UCA | -3.71 | -3.50 | -3.92 |
| 26 | Tajikistan | UCA | 2.52 | 2.75 | 2.28 |
| 27 | Republic of Moldova | UCA | -1.11 | -0.73 | -1.50 |
| 28 | Solomon Islands | UCA | -0.37 | -0.17 | -0.57 |
| 29 | Brazil | UCA | -1.54 | -1.24 | -1.83 |
| 30 | Russian Federation | UCA | -2.47 | -2.28 | -2.65 |
| 31 | United Arab Emirates | UCA | -3.03 | -2.71 | -3.34 |
| 32 | Tonga | UCA | -0.27 | -0.14 | -0.39 |
| 33 | Montenegro | UCA | -3.60 | -3.27 | -3.93 |
| 34 | Kuwait | UCA | 0.65 | 1.47 | -0.15 |
| 35 | Turkmenistan | UCA | 1.75 | 2.32 | 1.19 |
| 36 | Kenya | UCA | 0.17 | 0.34 | 0.00 |
| 37 | Yemen | UCA | -0.40 | -0.27 | -0.54 |
| 38 | Angola | UCA | -0.24 | -0.06 | -0.42 |
| 39 | Lebanon | UCA | -2.28 | -2.23 | -2.33 |
| 40 | Poland | UCA | -3.02 | -2.74 | -3.30 |
| 41 | Ukraine | UCA | -2.12 | -1.83 | -2.41 |
| 42 | Central African Republic | UCA | 0.20 | 0.30 | 0.10 |
| 43 | Andorra | UCA | -1.94 | -1.71 | -2.16 |
| 44 | Madagascar | UCA | -0.54 | -0.40 | -0.68 |
| 45 | Congo | UCA | -0.09 | -0.03 | -0.15 |
| 46 | Austria | UCA | -0.78 | -0.51 | -1.06 |
| 47 | Romania | UCA | -2.05 | -1.83 | -2.27 |
| 48 | Malawi | UCA | -1.17 | -0.94 | -1.41 |
| 49 | Chad | UCA | -0.22 | -0.13 | -0.31 |
| 50 | Israel | UCA | -2.78 | -2.43 | -3.13 |
| 51 | Sao Tome and Principe | UCA | -0.66 | -0.40 | -0.91 |
| 52 | Italy | UCA | -3.11 | -2.95 | -3.27 |
| 53 | Botswana | UCA | 0.84 | 0.96 | 0.72 |
| 54 | Lesotho | UCA | 0.63 | 0.76 | 0.51 |
| 55 | Honduras | UCA | 1.07 | 1.58 | 0.56 |
| 56 | Belize | UCA | -1.46 | -1.33 | -1.60 |
| 57 | Bermuda | UCA | -0.99 | -0.68 | -1.30 |
| 58 | Cuba | UCA | -2.19 | -2.01 | -2.37 |
| 59 | Mexico | UCA | -1.19 | -1.06 | -1.32 |
| 60 | Malaysia | UCA | 0.62 | 1.08 | 0.16 |
| 61 | Trinidad and Tobago | UCA | -1.97 | -1.56 | -2.38 |
| 62 | Puerto Rico | UCA | -3.45 | -2.94 | -3.96 |
| 63 | Armenia | UCA | 0.02 | 0.29 | -0.25 |
| 64 | Kiribati | UCA | -0.83 | -0.66 | -1.00 |
| 65 | Maldives | UCA | -1.79 | -1.50 | -2.09 |
| 66 | Marshall Islands | UCA | -0.16 | 0.15 | -0.47 |
| 67 | Lao People's Democratic Republic | UCA | -1.57 | -1.39 | -1.75 |
| 68 | Bhutan | UCA | -1.46 | -1.33 | -1.60 |
| 69 | Bahrain | UCA | -0.19 | 0.52 | -0.90 |
| 70 | Kazakhstan | UCA | -1.99 | -1.46 | -2.52 |
| 71 | Syrian Arab Republic | UCA | -2.70 | -1.88 | -3.52 |
| 72 | Myanmar | UCA | -0.38 | -0.06 | -0.69 |
| 73 | India | UCA | -0.65 | -0.55 | -0.75 |
| 74 | Azerbaijan | UCA | -0.48 | -0.23 | -0.72 |
| 75 | Belarus | UCA | -3.51 | -3.18 | -3.83 |
| 76 | Qatar | UCA | -2.88 | -2.77 | -2.99 |
| 77 | Bosnia and Herzegovina | UCA | -0.97 | -0.85 | -1.09 |
| 78 | Micronesia (Federated States of) | UCA | -1.10 | -1.01 | -1.18 |
| 79 | Nicaragua | UCA | -0.55 | -0.38 | -0.72 |
| 80 | Egypt | UCA | -2.69 | -2.39 | -2.99 |
| 81 | Latvia | UCA | -2.19 | -2.00 | -2.37 |
| 82 | Kyrgyzstan | UCA | -1.30 | -1.06 | -1.55 |
| 83 | Nepal | UCA | -0.87 | -0.70 | -1.04 |
| 84 | Papua New Guinea | UCA | 0.52 | 0.71 | 0.33 |
| 85 | Czechia | UCA | -3.14 | -2.76 | -3.53 |
| 86 | Iran (Islamic Republic of) | UCA | -2.69 | -2.35 | -3.02 |
| 87 | Georgia | UCA | 1.50 | 1.94 | 1.06 |
| 88 | Saudi Arabia | UCA | -4.46 | -4.30 | -4.61 |
| 89 | Tunisia | UCA | -3.21 | -3.13 | -3.30 |
| 90 | Republic of Korea | UCA | -1.11 | -0.83 | -1.40 |
| 91 | Pakistan | UCA | -0.37 | -0.16 | -0.58 |
| 92 | Djibouti | UCA | 0.21 | 0.34 | 0.09 |
| 93 | Lithuania | UCA | -3.04 | -2.77 | -3.31 |
| 94 | Mongolia | UCA | -0.84 | -0.32 | -1.35 |
| 95 | Hungary | UCA | -2.12 | -1.69 | -2.56 |
| 96 | Iraq | UCA | -2.68 | -2.34 | -3.02 |
| 97 | Australia | UCA | -1.84 | -1.67 | -2.00 |
| 98 | Turkey | UCA | -2.04 | -1.78 | -2.31 |
| 99 | Jordan | UCA | -2.35 | -2.21 | -2.49 |
| 100 | Eritrea | UCA | 0.23 | 0.35 | 0.11 |
| 101 | North Macedonia | UCA | -1.25 | -0.88 | -1.62 |
| 102 | Bulgaria | UCA | -2.21 | -2.00 | -2.41 |
| 103 | Estonia | UCA | -3.68 | -3.36 | -4.01 |
| 104 | Rwanda | UCA | -0.60 | -0.32 | -0.88 |
| 105 | United Republic of Tanzania | UCA | -0.12 | 0.06 | -0.29 |
| 106 | Singapore | UCA | -2.61 | -2.30 | -2.92 |
| 107 | Ethiopia | UCA | -1.27 | -1.12 | -1.42 |
| 108 | Burundi | UCA | -0.95 | -0.81 | -1.09 |
| 109 | Seychelles | UCA | 0.79 | 0.95 | 0.62 |
| 110 | Zimbabwe | UCA | 0.97 | 1.10 | 0.84 |
| 111 | Portugal | UCA | -2.87 | -2.59 | -3.15 |
| 112 | New Zealand | UCA | -2.52 | -2.28 | -2.75 |
| 113 | France | UCA | -1.30 | -1.09 | -1.51 |
| 114 | Uganda | UCA | 0.43 | 0.58 | 0.27 |
| 115 | Iceland | UCA | -2.23 | -1.92 | -2.54 |
| 116 | Mauritania | UCA | -0.31 | -0.21 | -0.42 |
| 117 | Zambia | UCA | -0.49 | -0.33 | -0.64 |
| 118 | Croatia | UCA | -0.73 | 0.00 | -1.45 |
| 119 | Switzerland | UCA | -3.69 | -3.52 | -3.86 |
| 120 | Spain | UCA | -3.28 | -3.15 | -3.42 |
| 121 | Burkina Faso | UCA | 0.54 | 0.67 | 0.41 |
| 122 | Somalia | UCA | 0.13 | 0.36 | -0.09 |
| 123 | Germany | UCA | -2.38 | -2.16 | -2.59 |
| 124 | Ireland | UCA | -2.73 | -2.44 | -3.02 |
| 125 | Guinea-Bissau | UCA | -0.85 | -0.75 | -0.95 |
| 126 | Comoros | UCA | -0.35 | -0.21 | -0.48 |
| 127 | Slovenia | UCA | -2.59 | -2.41 | -2.77 |
| 128 | Equatorial Guinea | UCA | -0.16 | 0.01 | -0.33 |
| 129 | Mauritius | UCA | -1.46 | -1.04 | -1.89 |
| 130 | Cambodia | UCA | -1.38 | -1.19 | -1.58 |
| 131 | American Samoa | UCA | 0.90 | 1.08 | 0.71 |
| 132 | Niger | UCA | -0.76 | -0.57 | -0.95 |
| 133 | United Kingdom | UCA | -1.68 | -1.56 | -1.79 |
| 134 | Cameroon | UCA | 0.36 | 0.47 | 0.24 |
| 135 | Greece | UCA | -1.59 | -1.29 | -1.90 |
| 136 | Sweden | UCA | -2.66 | -2.40 | -2.92 |
| 137 | Gabon | UCA | -0.34 | -0.16 | -0.52 |
| 138 | Malta | UCA | -0.75 | -0.46 | -1.04 |
| 139 | Palau | UCA | -0.71 | -0.62 | -0.80 |
| 140 | Liberia | UCA | -2.43 | -2.05 | -2.81 |
| 141 | Nigeria | UCA | 0.39 | 0.48 | 0.29 |
| 142 | Cabo Verde | UCA | -0.99 | -0.90 | -1.07 |
| 143 | Suriname | UCA | 0.09 | 0.32 | -0.14 |
| 144 | Argentina | UCA | -0.20 | 0.04 | -0.44 |
| 145 | Benin | UCA | 0.01 | 0.14 | -0.11 |
| 146 | Antigua and Barbuda | UCA | -0.10 | 0.00 | -0.19 |
| 147 | Indonesia | UCA | -1.09 | -1.03 | -1.16 |
| 148 | Barbados | UCA | -0.16 | 0.07 | -0.39 |
| 149 | Cyprus | UCA | -2.36 | -1.91 | -2.80 |
| 150 | Nauru | UCA | 0.75 | 1.29 | 0.22 |
| 151 | Brunei Darussalam | UCA | -0.70 | -0.55 | -0.84 |
| 152 | Mali | UCA | -0.14 | 0.06 | -0.33 |
| 153 | Bahamas | UCA | -1.08 | -0.95 | -1.20 |
| 154 | Namibia | UCA | 0.50 | 0.61 | 0.39 |
| 155 | Niue | UCA | 0.24 | 0.47 | 0.01 |
| 156 | Fiji | UCA | -0.15 | 0.13 | -0.43 |
| 157 | South Sudan | UCA | -0.04 | 0.23 | -0.31 |
| 158 | Netherlands | UCA | -4.03 | -3.74 | -4.32 |
| 159 | El Salvador | UCA | 4.02 | 5.27 | 2.79 |
| 160 | Northern Mariana Islands | UCA | 1.52 | 1.85 | 1.19 |
| 161 | Denmark | UCA | -4.95 | -4.47 | -5.42 |
| 162 | Jamaica | UCA | -1.16 | -0.86 | -1.45 |
| 163 | Gambia | UCA | -0.52 | -0.41 | -0.64 |
| 164 | Guatemala | UCA | -0.51 | -0.21 | -0.81 |
| 165 | Mozambique | UCA | -0.19 | -0.06 | -0.32 |
| 166 | Japan | UCA | -2.96 | -2.83 | -3.09 |
| 167 | South Africa | UCA | 0.72 | 0.83 | 0.61 |
| 168 | Saint Lucia | UCA | -0.10 | 0.04 | -0.24 |
| 169 | Finland | UCA | -4.16 | -3.90 | -4.41 |
| 170 | Algeria | UCA | -2.04 | -1.97 | -2.11 |
| 171 | Canada | UCA | -1.06 | -0.85 | -1.27 |
| 172 | Saint Vincent and the Grenadines | UCA | -1.65 | -1.51 | -1.79 |
| 173 | Grenada | UCA | -0.04 | 0.08 | -0.17 |
| 174 | Oman | UCA | -1.39 | -0.78 | -1.99 |
| 175 | Norway | UCA | -1.77 | -1.51 | -2.03 |
| 176 | Ghana | UCA | 0.77 | 0.90 | 0.63 |
| 177 | United States of America | UCA | -1.13 | -1.03 | -1.23 |
| 178 | Togo | UCA | -0.70 | -0.64 | -0.76 |
| 179 | Eswatini | UCA | 0.76 | 0.95 | 0.57 |
| 180 | Guyana | UCA | -0.44 | -0.20 | -0.68 |
| 181 | Democratic People's Republic of Korea | UCA | -2.64 | -2.56 | -2.72 |
| 182 | Guinea | UCA | -0.78 | -0.70 | -0.85 |
| 183 | Colombia | UCA | 0.42 | 0.87 | -0.02 |
| 184 | Tuvalu | UCA | -2.61 | -2.57 | -2.65 |
| 185 | Haiti | UCA | -0.46 | -0.24 | -0.69 |
| 186 | Taiwan (Province of China) | UCA | -1.07 | -0.70 | -1.44 |
| 187 | Libya | UCA | -2.96 | -2.80 | -3.12 |
| 188 | Timor-Leste | UCA | -0.98 | -0.92 | -1.04 |
| 189 | United States Virgin Islands | UCA | -2.47 | -2.34 | -2.60 |
| 190 | Monaco | UCA | -1.21 | -1.09 | -1.33 |
| 191 | Costa Rica | UCA | -1.20 | -0.92 | -1.48 |
| 192 | Uzbekistan | UCA | -0.58 | -0.45 | -0.71 |
| 193 | Paraguay | UCA | -0.43 | -0.07 | -0.79 |
| 194 | Viet Nam | UCA | -1.01 | -0.95 | -1.06 |
| 195 | Vanuatu | UCA | 0.29 | 0.51 | 0.07 |
| 196 | Morocco | UCA | -1.26 | -1.09 | -1.44 |
| 197 | Democratic Republic of the Congo | UCA | -0.63 | -0.50 | -0.76 |
| 198 | Palestine | UCA | -2.76 | -2.15 | -3.37 |
| 199 | Albania | UCA | -0.78 | -0.35 | -1.21 |
| 200 | Afghanistan | UCA | -1.45 | -1.27 | -1.62 |
| 201 | Sudan | UCA | -1.04 | -0.91 | -1.17 |
| 202 | Bangladesh | UCA | -1.34 | -1.25 | -1.42 |
| 203 | Serbia | UCA | -2.80 | -2.54 | -3.06 |
| 204 | Slovakia | UCA | -1.36 | -0.98 | -1.75 |
| 205 | Argentina | TS | 0.09 | 0.13 | 0.06 |
| 206 | Luxembourg | TS | -0.44 | -0.36 | -0.53 |
| 207 | Belgium | TS | -0.22 | -0.16 | -0.27 |
| 208 | Saint Helena | TS | 0.14 | 0.17 | 0.11 |
| 209 | Cuba | TS | -0.39 | -0.34 | -0.43 |
| 210 | Chile | TS | -0.45 | -0.41 | -0.49 |
| 211 | Uruguay | TS | 0.11 | 0.15 | 0.08 |
| 212 | Saint Kitts and Nevis | TS | -0.53 | -0.49 | -0.56 |
| 213 | Cook Islands | TS | 0.42 | 0.47 | 0.38 |
| 214 | Dominica | TS | -0.05 | 0.00 | -0.10 |
| 215 | Myanmar | TS | -0.07 | 0.00 | -0.13 |
| 216 | San Marino | TS | -0.02 | 0.01 | -0.05 |
| 217 | Senegal | TS | 0.08 | 0.11 | 0.05 |
| 218 | Dominican Republic | TS | -0.47 | -0.44 | -0.50 |
| 219 | Bolivia (Plurinational State of) | TS | -0.57 | -0.51 | -0.62 |
| 220 | Greenland | TS | -0.14 | -0.11 | -0.16 |
| 221 | Guam | TS | 0.16 | 0.24 | 0.09 |
| 222 | Tokelau | TS | -0.06 | -0.01 | -0.11 |
| 223 | Ecuador | TS | -0.53 | -0.49 | -0.57 |
| 224 | Philippines | TS | 0.22 | 0.25 | 0.20 |
| 225 | Panama | TS | -0.22 | -0.16 | -0.29 |
| 226 | Samoa | TS | 0.37 | 0.42 | 0.32 |
| 227 | Sri Lanka | TS | 0.40 | 0.46 | 0.35 |
| 228 | Venezuela (Bolivarian Republic of) | TS | -0.55 | -0.50 | -0.61 |
| 229 | Mongolia | TS | -0.30 | -0.24 | -0.35 |
| 230 | Turkey | TS | -1.54 | -1.39 | -1.70 |
| 231 | Tajikistan | TS | -0.05 | 0.01 | -0.11 |
| 232 | Republic of Moldova | TS | -0.07 | 0.03 | -0.18 |
| 233 | Jordan | TS | -1.08 | -0.98 | -1.19 |
| 234 | Solomon Islands | TS | 0.25 | 0.29 | 0.20 |
| 235 | North Macedonia | TS | -0.18 | -0.02 | -0.35 |
| 236 | Ethiopia | TS | -0.25 | -0.21 | -0.29 |
| 237 | United Arab Emirates | TS | -0.53 | -0.46 | -0.59 |
| 238 | Russian Federation | TS | -0.32 | -0.26 | -0.39 |
| 239 | Turkmenistan | TS | -0.31 | -0.26 | -0.36 |
| 240 | Montenegro | TS | 0.24 | 0.28 | 0.20 |
| 241 | Kuwait | TS | -0.89 | -0.80 | -0.99 |
| 242 | Kenya | TS | 0.36 | 0.39 | 0.33 |
| 243 | Yemen | TS | -0.80 | -0.74 | -0.87 |
| 244 | Angola | TS | -0.24 | -0.21 | -0.27 |
| 245 | Poland | TS | -0.38 | -0.24 | -0.53 |
| 246 | Ukraine | TS | 0.02 | 0.15 | -0.12 |
| 247 | Lebanon | TS | -1.06 | -0.96 | -1.16 |
| 248 | Central African Republic | TS | -0.01 | 0.04 | -0.06 |
| 249 | Madagascar | TS | 0.20 | 0.26 | 0.14 |
| 250 | Andorra | TS | -0.11 | -0.03 | -0.18 |
| 251 | Zambia | TS | -0.26 | -0.19 | -0.33 |
| 252 | Cabo Verde | TS | 0.21 | 0.28 | 0.14 |
| 253 | Austria | TS | -0.10 | -0.05 | -0.16 |
| 254 | Chad | TS | 0.06 | 0.11 | 0.00 |
| 255 | Nigeria | TS | -0.02 | 0.05 | -0.10 |
| 256 | Israel | TS | -0.37 | -0.35 | -0.40 |
| 257 | Sao Tome and Principe | TS | -0.22 | -0.16 | -0.28 |
| 258 | Italy | TS | -0.34 | -0.27 | -0.41 |
| 259 | Botswana | TS | -0.31 | -0.18 | -0.43 |
| 260 | Honduras | TS | -0.40 | -0.36 | -0.43 |
| 261 | Belize | TS | -0.31 | -0.24 | -0.39 |
| 262 | Lao People's Democratic Republic | TS | 0.19 | 0.25 | 0.13 |
| 263 | Trinidad and Tobago | TS | -0.51 | -0.43 | -0.59 |
| 264 | Mexico | TS | 0.41 | 0.45 | 0.37 |
| 265 | Malaysia | TS | 0.41 | 0.48 | 0.34 |
| 266 | Puerto Rico | TS | -0.25 | -0.22 | -0.29 |
| 267 | Georgia | TS | 0.10 | 0.26 | -0.06 |
| 268 | Saudi Arabia | TS | -1.23 | -1.15 | -1.31 |
| 269 | Armenia | TS | -0.51 | -0.40 | -0.62 |
| 270 | Kiribati | TS | 0.27 | 0.32 | 0.23 |
| 271 | Maldives | TS | 0.36 | 0.46 | 0.26 |
| 272 | Bangladesh | TS | -0.10 | -0.08 | -0.12 |
| 273 | Marshall Islands | TS | 0.23 | 0.27 | 0.19 |
| 274 | Slovenia | TS | -0.27 | -0.13 | -0.41 |
| 275 | Bhutan | TS | -0.40 | -0.35 | -0.46 |
| 276 | Egypt | TS | -0.73 | -0.66 | -0.79 |
| 277 | Bahrain | TS | -1.23 | -1.12 | -1.35 |
| 278 | Kazakhstan | TS | -0.65 | -0.54 | -0.75 |
| 279 | Estonia | TS | -0.64 | -0.48 | -0.80 |
| 280 | Syrian Arab Republic | TS | -0.97 | -0.83 | -1.10 |
| 281 | Nicaragua | TS | -0.82 | -0.71 | -0.92 |
| 282 | India | TS | 0.16 | 0.17 | 0.14 |
| 283 | Azerbaijan | TS | -0.33 | -0.28 | -0.37 |
| 284 | Belarus | TS | -0.30 | -0.25 | -0.36 |
| 285 | Bosnia and Herzegovina | TS | -0.35 | -0.14 | -0.57 |
| 286 | Qatar | TS | -1.26 | -1.10 | -1.42 |
| 287 | Micronesia (Federated States of) | TS | 0.19 | 0.23 | 0.15 |
| 288 | Comoros | TS | -0.19 | -0.16 | -0.22 |
| 289 | Nepal | TS | 0.04 | 0.09 | -0.01 |
| 290 | Latvia | TS | -0.27 | -0.12 | -0.41 |
| 291 | Kyrgyzstan | TS | -0.30 | -0.25 | -0.36 |
| 292 | Tunisia | TS | -0.93 | -0.84 | -1.01 |
| 293 | Gabon | TS | -0.07 | -0.03 | -0.11 |
| 294 | Papua New Guinea | TS | 0.29 | 0.33 | 0.24 |
| 295 | Czechia | TS | -0.11 | 0.05 | -0.27 |
| 296 | Iran (Islamic Republic of) | TS | -0.72 | -0.65 | -0.78 |
| 297 | Republic of Korea | TS | -0.79 | -0.72 | -0.87 |
| 298 | Pakistan | TS | 0.23 | 0.27 | 0.19 |
| 299 | Lithuania | TS | 0.14 | 0.29 | -0.02 |
| 300 | Rwanda | TS | -0.54 | -0.48 | -0.60 |
| 301 | Djibouti | TS | -0.29 | -0.22 | -0.36 |
| 302 | Iraq | TS | -0.82 | -0.73 | -0.91 |
| 303 | Hungary | TS | 0.01 | 0.21 | -0.19 |
| 304 | Eritrea | TS | -0.08 | -0.04 | -0.11 |
| 305 | Australia | TS | -0.09 | -0.04 | -0.14 |
| 306 | Eswatini | TS | 0.24 | 0.38 | 0.11 |
| 307 | Bulgaria | TS | 0.14 | 0.22 | 0.06 |
| 308 | Finland | TS | -0.35 | -0.32 | -0.38 |
| 309 | United Republic of Tanzania | TS | 0.07 | 0.12 | 0.02 |
| 310 | Singapore | TS | -0.97 | -0.86 | -1.09 |
| 311 | Greece | TS | 0.00 | 0.06 | -0.06 |
| 312 | Burundi | TS | -0.07 | -0.02 | -0.12 |
| 313 | Seychelles | TS | 0.28 | 0.36 | 0.20 |
| 314 | Portugal | TS | -0.64 | -0.60 | -0.68 |
| 315 | Zimbabwe | TS | 0.58 | 0.76 | 0.40 |
| 316 | France | TS | -0.39 | -0.35 | -0.44 |
| 317 | New Zealand | TS | 0.10 | 0.13 | 0.07 |
| 318 | Mali | TS | -0.02 | 0.07 | -0.11 |
| 319 | Uganda | TS | -0.03 | 0.00 | -0.07 |
| 320 | Benin | TS | 0.08 | 0.13 | 0.04 |
| 321 | Iceland | TS | 0.03 | 0.11 | -0.05 |
| 322 | Mauritania | TS | -0.25 | -0.20 | -0.30 |
| 323 | Croatia | TS | -0.15 | 0.01 | -0.30 |
| 324 | Switzerland | TS | -0.16 | -0.12 | -0.20 |
| 325 | Ireland | TS | -0.65 | -0.60 | -0.71 |
| 326 | Spain | TS | -0.44 | -0.38 | -0.49 |
| 327 | Burkina Faso | TS | 0.07 | 0.15 | -0.01 |
| 328 | Somalia | TS | -0.03 | 0.00 | -0.05 |
| 329 | Germany | TS | -0.17 | -0.10 | -0.23 |
| 330 | Guinea-Bissau | TS | 0.02 | 0.06 | -0.02 |
| 331 | Northern Mariana Islands | TS | 0.30 | 0.36 | 0.24 |
| 332 | Niger | TS | -0.02 | 0.02 | -0.06 |
| 333 | Lesotho | TS | 0.13 | 0.26 | 0.00 |
| 334 | Cambodia | TS | -0.09 | -0.03 | -0.15 |
| 335 | Equatorial Guinea | TS | -1.14 | -1.01 | -1.27 |
| 336 | Mauritius | TS | 0.10 | 0.16 | 0.03 |
| 337 | American Samoa | TS | 0.43 | 0.48 | 0.39 |
| 338 | Cameroon | TS | -0.06 | -0.02 | -0.09 |
| 339 | Monaco | TS | 0.10 | 0.13 | 0.07 |
| 340 | United Kingdom | TS | -0.10 | -0.08 | -0.12 |
| 341 | Sweden | TS | 0.08 | 0.12 | 0.03 |
| 342 | Malta | TS | -0.22 | -0.20 | -0.24 |
| 343 | Saint Vincent and the Grenadines | TS | -0.23 | -0.17 | -0.30 |
| 344 | Bermuda | TS | -0.28 | -0.22 | -0.34 |
| 345 | Palau | TS | 0.41 | 0.48 | 0.35 |
| 346 | Liberia | TS | -0.10 | -0.01 | -0.18 |
| 347 | Suriname | TS | -0.34 | -0.29 | -0.40 |
| 348 | Bahamas | TS | -0.29 | -0.24 | -0.35 |
| 349 | Antigua and Barbuda | TS | -0.46 | -0.39 | -0.54 |
| 350 | Indonesia | TS | 0.02 | 0.06 | -0.01 |
| 351 | Barbados | TS | -0.44 | -0.39 | -0.48 |
| 352 | Sudan | TS | -0.82 | -0.75 | -0.89 |
| 353 | Cyprus | TS | -0.87 | -0.80 | -0.95 |
| 354 | Nauru | TS | 0.46 | 0.62 | 0.31 |
| 355 | Brunei Darussalam | TS | -0.29 | -0.24 | -0.34 |
| 356 | Namibia | TS | -0.36 | -0.24 | -0.48 |
| 357 | Mozambique | TS | -0.05 | -0.02 | -0.08 |
| 358 | Costa Rica | TS | -0.53 | -0.48 | -0.58 |
| 359 | Niue | TS | 0.54 | 0.61 | 0.47 |
| 360 | Fiji | TS | 0.31 | 0.36 | 0.26 |
| 361 | South Sudan | TS | 0.25 | 0.29 | 0.21 |
| 362 | Netherlands | TS | -0.32 | -0.24 | -0.40 |
| 363 | El Salvador | TS | -0.76 | -0.71 | -0.81 |
| 364 | Jamaica | TS | -0.30 | -0.21 | -0.38 |
| 365 | Denmark | TS | -0.21 | -0.16 | -0.25 |
| 366 | Gambia | TS | 0.18 | 0.21 | 0.15 |
| 367 | Japan | TS | 0.11 | 0.13 | 0.10 |
| 368 | Guatemala | TS | -0.29 | -0.26 | -0.31 |
| 369 | Sierra Leone | TS | 0.06 | 0.09 | 0.04 |
| 370 | South Africa | TS | 0.08 | 0.26 | -0.09 |
| 371 | Saint Lucia | TS | -0.51 | -0.47 | -0.55 |
| 372 | Algeria | TS | -0.84 | -0.77 | -0.92 |
| 373 | Canada | TS | -0.63 | -0.59 | -0.67 |
| 374 | Grenada | TS | -0.39 | -0.36 | -0.42 |
| 375 | China | TS | 0.29 | 0.33 | 0.25 |
| 376 | Oman | TS | -0.98 | -0.86 | -1.10 |
| 377 | Peru | TS | -0.88 | -0.81 | -0.96 |
| 378 | Norway | TS | 0.09 | 0.10 | 0.08 |
| 379 | Ghana | TS | 0.12 | 0.18 | 0.07 |
| 380 | United States of America | TS | -0.13 | -0.12 | -0.14 |
| 381 | Togo | TS | 0.15 | 0.18 | 0.12 |
| 382 | Guinea | TS | 0.14 | 0.18 | 0.10 |
| 383 | Guyana | TS | -0.42 | -0.36 | -0.48 |
| 384 | Democratic People's Republic of Korea | TS | 0.83 | 1.04 | 0.63 |
| 385 | Thailand | TS | 0.48 | 0.57 | 0.39 |
| 386 | Colombia | TS | -0.70 | -0.66 | -0.73 |
| 387 | Tuvalu | TS | 0.24 | 0.28 | 0.20 |
| 388 | Haiti | TS | -0.37 | -0.32 | -0.41 |
| 389 | Brazil | TS | -0.13 | -0.11 | -0.15 |
| 390 | Taiwan (Province of China) | TS | 0.04 | 0.10 | -0.02 |
| 391 | Libya | TS | -0.70 | -0.55 | -0.85 |
| 392 | Timor-Leste | TS | -0.06 | -0.02 | -0.11 |
| 393 | United States Virgin Islands | TS | -0.47 | -0.40 | -0.55 |
| 394 | Uzbekistan | TS | -0.15 | -0.10 | -0.19 |
| 395 | Tonga | TS | 0.35 | 0.40 | 0.31 |
| 396 | Paraguay | TS | -0.58 | -0.49 | -0.66 |
| 397 | Viet Nam | TS | 0.08 | 0.15 | 0.01 |
| 398 | Congo | TS | -0.12 | -0.06 | -0.17 |
| 399 | Vanuatu | TS | 0.23 | 0.27 | 0.19 |
| 400 | Morocco | TS | -0.80 | -0.72 | -0.88 |
| 401 | Democratic Republic of the Congo | TS | 0.22 | 0.25 | 0.18 |
| 402 | Romania | TS | -0.26 | -0.11 | -0.42 |
| 403 | Palestine | TS | -0.44 | -0.39 | -0.49 |
| 404 | Albania | TS | -0.31 | -0.17 | -0.45 |
| 405 | Afghanistan | TS | -0.83 | -0.75 | -0.91 |
| 406 | Malawi | TS | 0.01 | 0.05 | -0.03 |
| 407 | Serbia | TS | -0.32 | -0.21 | -0.43 |
| 408 | Slovakia | TS | 0.00 | 0.10 | -0.09 |
| 409 | Argentina | KS | -0.47 | -0.34 | -0.60 |
| 410 | Luxembourg | KS | -0.14 | -0.05 | -0.22 |
| 411 | Belgium | KS | -0.34 | -0.27 | -0.41 |
| 412 | Saint Helena | KS | -0.28 | -0.22 | -0.34 |
| 413 | Cuba | KS | -0.04 | 0.03 | -0.10 |
| 414 | Chile | KS | -0.27 | -0.14 | -0.40 |
| 415 | Uruguay | KS | -0.52 | -0.41 | -0.64 |
| 416 | Saint Kitts and Nevis | KS | -0.10 | -0.05 | -0.15 |
| 417 | Cook Islands | KS | 0.18 | 0.21 | 0.15 |
| 418 | Dominica | KS | 0.13 | 0.21 | 0.06 |
| 419 | Myanmar | KS | 0.02 | 0.04 | -0.01 |
| 420 | San Marino | KS | 0.22 | 0.26 | 0.18 |
| 421 | Senegal | KS | -0.18 | -0.14 | -0.22 |
| 422 | Dominican Republic | KS | 0.01 | 0.06 | -0.04 |
| 423 | Bolivia (Plurinational State of) | KS | -0.75 | -0.68 | -0.81 |
| 424 | Greenland | KS | 0.08 | 0.11 | 0.05 |
| 425 | Guam | KS | 0.10 | 0.14 | 0.06 |
| 426 | Tokelau | KS | -0.13 | -0.09 | -0.16 |
| 427 | Ecuador | KS | -0.56 | -0.46 | -0.65 |
| 428 | Philippines | KS | 0.08 | 0.11 | 0.05 |
| 429 | Panama | KS | -0.09 | 0.01 | -0.19 |
| 430 | Samoa | KS | 0.17 | 0.20 | 0.14 |
| 431 | Sri Lanka | KS | 0.19 | 0.22 | 0.15 |
| 432 | Venezuela (Bolivarian Republic of) | KS | -0.27 | -0.23 | -0.30 |
| 433 | Mongolia | KS | 0.09 | 0.25 | -0.06 |
| 434 | Turkey | KS | 0.44 | 0.56 | 0.31 |
| 435 | Tajikistan | KS | 0.02 | 0.16 | -0.12 |
| 436 | Republic of Moldova | KS | 0.05 | 0.52 | -0.42 |
| 437 | Jordan | KS | 0.16 | 0.27 | 0.05 |
| 438 | North Macedonia | KS | 0.25 | 0.45 | 0.04 |
| 439 | Solomon Islands | KS | 0.16 | 0.20 | 0.12 |
| 440 | Ethiopia | KS | 0.03 | 0.11 | -0.04 |
| 441 | Russian Federation | KS | 0.14 | 0.62 | -0.34 |
| 442 | United Arab Emirates | KS | 0.03 | 0.12 | -0.06 |
| 443 | Turkmenistan | KS | -0.06 | 0.09 | -0.20 |
| 444 | Montenegro | KS | 0.67 | 0.94 | 0.39 |
| 445 | Kuwait | KS | -0.07 | 0.00 | -0.15 |
| 446 | Kenya | KS | -0.23 | -0.19 | -0.27 |
| 447 | Yemen | KS | 0.18 | 0.30 | 0.07 |
| 448 | Angola | KS | 0.01 | 0.06 | -0.03 |
| 449 | Poland | KS | 0.51 | 0.89 | 0.14 |
| 450 | Ukraine | KS | 0.15 | 0.62 | -0.31 |
| 451 | Lebanon | KS | 0.00 | 0.09 | -0.08 |
| 452 | Central African Republic | KS | -0.06 | 0.00 | -0.12 |
| 453 | Madagascar | KS | -0.20 | -0.15 | -0.26 |
| 454 | Andorra | KS | 0.26 | 0.38 | 0.14 |
| 455 | Zambia | KS | 0.10 | 0.18 | 0.02 |
| 456 | Cabo Verde | KS | -0.12 | -0.09 | -0.16 |
| 457 | Austria | KS | 0.34 | 0.39 | 0.29 |
| 458 | Nigeria | KS | 0.03 | 0.06 | 0.00 |
| 459 | Chad | KS | -0.13 | -0.09 | -0.16 |
| 460 | Israel | KS | -0.17 | -0.11 | -0.22 |
| 461 | Sao Tome and Principe | KS | -0.01 | 0.04 | -0.05 |
| 462 | Italy | KS | 0.72 | 0.81 | 0.64 |
| 463 | Botswana | KS | 0.13 | 0.20 | 0.07 |
| 464 | Honduras | KS | -0.21 | -0.17 | -0.26 |
| 465 | Belize | KS | -0.01 | 0.05 | -0.08 |
| 466 | Lao People's Democratic Republic | KS | -0.01 | 0.02 | -0.05 |
| 467 | Mexico | KS | -0.24 | -0.19 | -0.28 |
| 468 | Malaysia | KS | 0.17 | 0.20 | 0.14 |
| 469 | Puerto Rico | KS | 0.04 | 0.11 | -0.02 |
| 470 | Trinidad and Tobago | KS | -0.02 | 0.05 | -0.09 |
| 471 | Georgia | KS | 0.11 | 0.29 | -0.07 |
| 472 | Saudi Arabia | KS | 0.32 | 0.42 | 0.23 |
| 473 | Armenia | KS | -0.04 | 0.10 | -0.17 |
| 474 | Kiribati | KS | 0.10 | 0.13 | 0.06 |
| 475 | Maldives | KS | 0.16 | 0.19 | 0.12 |
| 476 | Marshall Islands | KS | 0.15 | 0.19 | 0.11 |
| 477 | Bangladesh | KS | 0.07 | 0.10 | 0.04 |
| 478 | Slovenia | KS | 0.18 | 0.31 | 0.05 |
| 479 | Bhutan | KS | 0.25 | 0.29 | 0.21 |
| 480 | Egypt | KS | 0.15 | 0.26 | 0.04 |
| 481 | Bahrain | KS | 0.07 | 0.18 | -0.03 |
| 482 | Kazakhstan | KS | -0.11 | 0.02 | -0.23 |
| 483 | Estonia | KS | 0.14 | 0.57 | -0.29 |
| 484 | Syrian Arab Republic | KS | 0.32 | 0.47 | 0.17 |
| 485 | Nicaragua | KS | -0.26 | -0.20 | -0.33 |
| 486 | India | KS | -0.05 | -0.02 | -0.07 |
| 487 | Azerbaijan | KS | -0.02 | 0.11 | -0.14 |
| 488 | Belarus | KS | 0.14 | 0.57 | -0.30 |
| 489 | Qatar | KS | -0.04 | 0.04 | -0.13 |
| 490 | Bosnia and Herzegovina | KS | 0.12 | 0.29 | -0.05 |
| 491 | Micronesia (Federated States of) | KS | 0.07 | 0.10 | 0.04 |
| 492 | Comoros | KS | 0.09 | 0.13 | 0.05 |
| 493 | Nepal | KS | -0.17 | -0.11 | -0.23 |
| 494 | Latvia | KS | 0.20 | 0.62 | -0.23 |
| 495 | Kyrgyzstan | KS | -0.01 | 0.13 | -0.14 |
| 496 | Tunisia | KS | 0.14 | 0.23 | 0.05 |
| 497 | Papua New Guinea | KS | 0.15 | 0.18 | 0.12 |
| 498 | Czechia | KS | 0.82 | 1.35 | 0.30 |
| 499 | Iran (Islamic Republic of) | KS | 0.04 | 0.11 | -0.03 |
| 500 | Gabon | KS | 0.06 | 0.09 | 0.04 |
| 501 | Republic of Korea | KS | 0.77 | 0.90 | 0.63 |
| 502 | Pakistan | KS | -0.17 | -0.14 | -0.20 |
| 503 | Lithuania | KS | 0.32 | 0.72 | -0.09 |
| 504 | Rwanda | KS | 0.20 | 0.25 | 0.15 |
| 505 | Djibouti | KS | 0.09 | 0.15 | 0.03 |
| 506 | Hungary | KS | 1.42 | 1.84 | 1.00 |
| 507 | Iraq | KS | 0.14 | 0.26 | 0.01 |
| 508 | Eritrea | KS | -0.16 | -0.11 | -0.21 |
| 509 | Australia | KS | 0.42 | 0.56 | 0.27 |
| 510 | Eswatini | KS | -0.29 | -0.20 | -0.38 |
| 511 | Bulgaria | KS | 0.69 | 0.96 | 0.42 |
| 512 | Finland | KS | -0.18 | -0.13 | -0.23 |
| 513 | United Republic of Tanzania | KS | -0.12 | -0.07 | -0.17 |
| 514 | Singapore | KS | 0.53 | 0.61 | 0.45 |
| 515 | Greece | KS | 0.43 | 0.50 | 0.36 |
| 516 | Burundi | KS | -0.06 | -0.02 | -0.10 |
| 517 | Seychelles | KS | 0.14 | 0.17 | 0.11 |
| 518 | Portugal | KS | -0.45 | -0.30 | -0.60 |
| 519 | Zimbabwe | KS | -0.38 | -0.24 | -0.51 |
| 520 | France | KS | 0.08 | 0.17 | -0.01 |
| 521 | New Zealand | KS | 0.09 | 0.13 | 0.06 |
| 522 | Mali | KS | -0.19 | -0.12 | -0.27 |
| 523 | Uganda | KS | -0.04 | 0.00 | -0.07 |
| 524 | Benin | KS | -0.29 | -0.24 | -0.35 |
| 525 | Iceland | KS | 0.19 | 0.29 | 0.10 |
| 526 | Mauritania | KS | 0.03 | 0.08 | -0.02 |
| 527 | Croatia | KS | 0.05 | 0.30 | -0.20 |
| 528 | Switzerland | KS | 0.26 | 0.40 | 0.12 |
| 529 | Ireland | KS | -0.21 | -0.15 | -0.26 |
| 530 | Spain | KS | 0.24 | 0.28 | 0.19 |
| 531 | Burkina Faso | KS | -0.23 | -0.19 | -0.27 |
| 532 | Somalia | KS | -0.07 | -0.01 | -0.14 |
| 533 | Germany | KS | 0.20 | 0.26 | 0.15 |
| 534 | Guinea-Bissau | KS | -0.05 | -0.01 | -0.08 |
| 535 | Northern Mariana Islands | KS | 0.11 | 0.14 | 0.08 |
| 536 | Niger | KS | -0.20 | -0.16 | -0.24 |
| 537 | Lesotho | KS | -0.02 | 0.04 | -0.07 |
| 538 | Cambodia | KS | -0.19 | -0.15 | -0.23 |
| 539 | Equatorial Guinea | KS | 0.39 | 0.44 | 0.33 |
| 540 | Mauritius | KS | 0.03 | 0.07 | 0.00 |
| 541 | American Samoa | KS | 0.20 | 0.23 | 0.17 |
| 542 | Cameroon | KS | -0.12 | -0.06 | -0.19 |
| 543 | United Kingdom | KS | 0.14 | 0.16 | 0.11 |
| 544 | Sweden | KS | 0.41 | 0.52 | 0.30 |
| 545 | Malta | KS | -0.07 | 0.00 | -0.14 |
| 546 | Saint Vincent and the Grenadines | KS | 0.08 | 0.14 | 0.01 |
| 547 | Bermuda | KS | 0.26 | 0.33 | 0.19 |
| 548 | Palau | KS | 0.20 | 0.23 | 0.18 |
| 549 | Liberia | KS | -0.05 | -0.01 | -0.10 |
| 550 | Suriname | KS | -0.01 | 0.04 | -0.07 |
| 551 | Bahamas | KS | 0.11 | 0.17 | 0.05 |
| 552 | Monaco | KS | 0.32 | 0.35 | 0.28 |
| 553 | Antigua and Barbuda | KS | -0.06 | 0.02 | -0.15 |
| 554 | Indonesia | KS | 0.06 | 0.09 | 0.03 |
| 555 | Barbados | KS | -0.04 | 0.02 | -0.11 |
| 556 | Sudan | KS | 0.15 | 0.28 | 0.02 |
| 557 | Cyprus | KS | -0.44 | -0.37 | -0.50 |
| 558 | Nauru | KS | 0.13 | 0.15 | 0.10 |
| 559 | Brunei Darussalam | KS | 0.33 | 0.42 | 0.24 |
| 560 | Namibia | KS | 0.32 | 0.37 | 0.27 |
| 561 | Costa Rica | KS | -0.34 | -0.30 | -0.38 |
| 562 | Niue | KS | 0.34 | 0.37 | 0.32 |
| 563 | Fiji | KS | 0.13 | 0.17 | 0.10 |
| 564 | South Sudan | KS | -0.16 | -0.10 | -0.22 |
| 565 | Netherlands | KS | -0.17 | -0.07 | -0.26 |
| 566 | El Salvador | KS | -0.13 | -0.07 | -0.19 |
| 567 | Jamaica | KS | 0.12 | 0.18 | 0.07 |
| 568 | Denmark | KS | -0.35 | -0.29 | -0.41 |
| 569 | Gambia | KS | -0.28 | -0.22 | -0.33 |
| 570 | Guatemala | KS | -0.22 | -0.18 | -0.26 |
| 571 | Mozambique | KS | -0.04 | 0.01 | -0.09 |
| 572 | Sierra Leone | KS | -0.24 | -0.18 | -0.30 |
| 573 | Japan | KS | 0.44 | 0.50 | 0.38 |
| 574 | South Africa | KS | -0.09 | -0.02 | -0.15 |
| 575 | Saint Lucia | KS | 0.02 | 0.08 | -0.03 |
| 576 | Algeria | KS | 0.19 | 0.30 | 0.08 |
| 577 | Canada | KS | -0.33 | -0.28 | -0.37 |
| 578 | Grenada | KS | 0.06 | 0.11 | 0.01 |
| 579 | China | KS | -0.69 | -0.57 | -0.81 |
| 580 | Oman | KS | -0.03 | 0.05 | -0.10 |
| 581 | Peru | KS | -0.71 | -0.64 | -0.78 |
| 582 | Norway | KS | 0.09 | 0.14 | 0.04 |
| 583 | Ghana | KS | -0.19 | -0.13 | -0.25 |
| 584 | United States of America | KS | -0.12 | -0.08 | -0.15 |
| 585 | Togo | KS | -0.27 | -0.22 | -0.32 |
| 586 | Guinea | KS | -0.21 | -0.17 | -0.25 |
| 587 | Guyana | KS | -0.02 | 0.06 | -0.09 |
| 588 | Democratic People's Republic of Korea | KS | 0.32 | 0.42 | 0.23 |
| 589 | Thailand | KS | 0.19 | 0.22 | 0.15 |
| 590 | Colombia | KS | -0.50 | -0.42 | -0.58 |
| 591 | Tuvalu | KS | 0.03 | 0.06 | 0.00 |
| 592 | Brazil | KS | 0.96 | 1.02 | 0.90 |
| 593 | Haiti | KS | -0.07 | -0.01 | -0.14 |
| 594 | Taiwan (Province of China) | KS | 1.15 | 1.20 | 1.10 |
| 595 | Libya | KS | 0.18 | 0.30 | 0.05 |
| 596 | Timor-Leste | KS | -0.05 | -0.02 | -0.09 |
| 597 | United States Virgin Islands | KS | -0.11 | -0.05 | -0.17 |
| 598 | Uzbekistan | KS | 0.05 | 0.19 | -0.09 |
| 599 | Tonga | KS | 0.13 | 0.16 | 0.10 |
| 600 | Paraguay | KS | 0.41 | 0.51 | 0.31 |
| 601 | Viet Nam | KS | 0.11 | 0.16 | 0.06 |
| 602 | Congo | KS | 0.00 | 0.05 | -0.05 |
| 603 | Vanuatu | KS | 0.08 | 0.12 | 0.05 |
| 604 | Morocco | KS | 0.20 | 0.31 | 0.08 |
| 605 | Democratic Republic of the Congo | KS | -0.23 | -0.19 | -0.27 |
| 606 | Romania | KS | 0.23 | 0.43 | 0.02 |
| 607 | Palestine | KS | -0.09 | -0.04 | -0.15 |
| 608 | Albania | KS | -0.08 | 0.13 | -0.29 |
| 609 | Afghanistan | KS | 0.19 | 0.34 | 0.04 |
| 610 | Malawi | KS | -0.10 | -0.06 | -0.15 |
| 611 | Serbia | KS | 0.16 | 0.40 | -0.07 |
| 612 | Slovakia | KS | 0.58 | 0.81 | 0.36 |
